# A MYB–WRKY feedback module activates *MdAAT2‑like* to regulate aromatic ester biosynthesis during apple ripening

**DOI:** 10.1186/s43897-026-00240-z

**Published:** 2026-08-03

**Authors:** Bin Wang, Fang Zhou, Yingrui Gao, Min Cai, Xiaoxiao He, Lulong Sun, Zhengyang Zhao

**Affiliations:** State Key Laboratory of Crop Stress Biology for Arid Areas, College of Horticulture, Northwest A&F University, Yangling, Shaanxi 712100 China

**Keywords:** Aromatic esters, Apple fruit ripening, MdMYB98-like, MdWRKY21, Positive feedback loop

## Abstract

Aromatic esters are key determinants of apple fruit aroma and consumer preference. Here, we investigated the molecular mechanisms underlying ester biosynthesis by characterizing *MdAAT2-like*, a critical ester-synthesizing gene in apple. *MdAAT2-like* expressions were significantly upregulated during fruit ripening and positively correlated with ester accumulation. Functional validation in apple and tomato demonstrated that *MdAAT2-like* overexpression enhanced ester contents, while silencing or knockout reduced ester production. Enzymatic assays revealed that MdAAT2-like exhibits higher catalytic efficiency for medium- and short-chain acyl-CoAs compared to other AAT family members. Two transcription factors, MdMYB98-like and MdWRKY21, were identified as direct activators of *MdAAT2-like*. Both factors bind its promoter and synergistically enhance transcription through protein interaction. Notably, we uncovered a positive feedback model wherein *MdMYB98-like* and *MdWRKY21* reciprocally activate each other's expression, reinforcing rapid ester synthesis during ripening. This study reveals a MYB-WRKY regulatory module controlling ester biosynthesis in apple.

**Supplementary Information:**

The online version contains supplementary material available at 10.1186/s43897-026-00240-z.

## Core

This study reveals a MYB-WRKY feedback module controlling apple ester biosynthesis. MdMYB98-like and MdWRKY21 synergistically activate *MdAAT2-like* expression and reciprocally enhance each other's transcription, reinforcing ester synthesis during fruit ripening.

## Gene & accession numbers

The transcriptome date during development were derived from Liu et al. (2021) and are available in the NCBI database: the accession number is PRJNA728501. The sequence data used in this study can be found in the GenBank data libraries under the following accession numbers: *MdAAT2-like* (MD02G1015200), *MdMYB98-like* (MD06G1172900), *MdWRKY21* (MD10G1191400), *MdAAT1* (MD02G1013900), and *MdAAT2* (MD02G1014800).

## Introduction

Flavor is a key determinant of fruit quality, shaped by the balance of sugars, organic acids, and volatile compounds (Lin et al. 2025). Among these, aromatic compounds are particularly important, as they not only define the sensory attributes of apples but also influence consumer preference and market value. Improving apple aroma has therefore been a primary focus in breeding and postharvest research. Apple aroma is a complex mixture of volatile compounds, including aldehydes, alcohols, esters, terpenes, and ketones, with their composition and concentration varying across cultivars (Zhang et al. 2024a). Among these, esters are the predominant contributors to the characteristic fruity aroma of apples (Niu et al. 2019). However, their biosynthesis is regulated by complex metabolic pathways and influenced by the genetic diversity of apple cultivars, making trait optimization particularly challenging (Costa et al. 2011). To date, over 350 volatile compounds have been identified in apples, with esters accounting for more than 80% of the aroma-related components (Espino-Díaz et al. 2016). The biosynthesis of esters is closely associated with fruit ripening, during which the levels of alcohol and ester-derived aromatic substances progressively increase (Liu et al. 2021). As ripening advances, esters become the dominant contributors to apple aroma, underscoring their importance in fruit quality and sensory appeal.

In plants, ester biosynthesis primarily derives from fatty acid and amino acid metabolism, where precursor molecules serve as substrates for enzymatic conversion into volatile aromatic compounds. A key enzyme in this process is alcohol acyltransferase (AAT), which catalyzes the formation of aromatic esters by facilitating the transfer of acyl groups from acyl-CoA donors to alcohol acceptor molecules. AAT belongs to the BAHD acyltransferase family and plays a crucial role in converting alcohols produced through fatty acid and amino acid metabolism into aromatic esters (Li et al. 2014). Several AAT genes have been identified in fruit-bearing plants, including bananas, melons, and apples, as key contributors to ester biosynthesis (Beekwilder et al. 2004; El-Sharkawy et al. 2005; Goulet et al. 2015; Souleyre et al. 2014; Wang & Luca 2010). In apples, the genome contains 17 MdAAT genes, with *MdAAT1* playing a significant role in the production of esters, including hexyl acetate, butyl acetate, and 2-methylbutyl acetate in the ‘Royal Gala’ variety (Souleyre et al. 2005). Additionally, *MdAAT1* and *MdAAT2* have been identified as key contributors to the synthesis of fruit-derived aromatic esters (Li et al. 2006a; Souleyre et al. 2005). Consequently, AAT—a key rate-limiting enzyme—is essential for ester biosynthesis in apple fruit (Defilippi et al. 2005; Li et al. 2006b). Furthermore, exogenous ethylene and salicylic acid have been shown to significantly induce the expression of *MdAAT2*, enhancing ester biosynthesis in apples (Li et al. 2006a).

The regulatory role of transcription factors in the biosynthesis of aromatic compounds has been demonstrated in various plant species. Several key transcription factors involved in this process have been identified, including *MYC2* in *Arabidopsis* (Hong et al. 2012), *AaNAC2/3/4* in kiwi (Nieuwenhuizen et al. 2015), and *CitAP2.10* and *CitERF71* in citrus (Liu et al. 2017). In tomatoes, *SlRIN* has been shown to regulate the synthesis of aromatics through the fatty acid pathway (Qin et al. 2012), while *SlMYB75* enhances terpene volatile synthesis by activating TPS expression and promotes aldehyde volatile synthesis by binding to *SlLOXC* and *SlAADC2* in the lipoxygenase pathway (Jian et al. 2019). Recent studies in strawberries have identified transcription factors such as *FaEGS1*, *FaEGS2*, *FaCAD1*, *FaEOBII*, and *FaMYB10*, which directly activate genes involved in eugenol biosynthesis (Shuaishuai et al. 2022). In peaches, NAC transcription factors have been implicated in ester biosynthesis, with evidence from electrophoretic mobility shift assays (EMSAs) and bimolecular fluorescence complementation (BiFC) studies suggesting that PpNAC1 and PpNAC2 form dimers, bind to the *PpAAT1* promoter, and jointly regulate its expression, thereby influencing ester production (Zhang et al. 2024b). In apples, transcription factors such as MdMYB85 and MdMYC2 have been shown to positively regulate the activity of *MdAAT1* promoter, while MdMYB1 and MdMYB6 bind to the *MdAAT2* promoter and participate in regulatory pathways involving salicylic acid and ethylene (Li et al. 2014, 2023). These transcription factors play a crucial role in modulating the biosynthesis of esters, alcohols, aldehydes, and terpenes. However, most existing studies have focused on linear regulatory pathways controlled by individual transcription factors or members within the same family, while how transcription factors from different families (e.g., MYB and WRKY) fine-tune ester biosynthesis through cooperative interactions remains an unresolved question. Notably, the synergistic role of MYB-WRKY regulatory modules in other plant secondary metabolic pathways has been documented (Zhang et al. 2024c; Liu et al. 2022).

To explore such regulatory mechanisms in apple, we screened for MYB and WRKY transcription factors whose expression was tightly correlated with both the key ester synthesis gene *MdAAT2-like* and aromatic ester accumulation during fruit ripening. Through this approach, MdMYB98-like and MdWRKY21 were identified as high-priority candidates. Therefore, this study aims to elucidate the following questions: whether MdMYB98-like and MdWRKY21 physically interact individually and cooperatively regulate *MdAAT2-like* promoter activity; and whether a feedback regulatory loop exists between them, thereby revealing their synergistic regulatory mechanism in apple aromatic ester biosynthesis.

Through gene expression analysis, genetic transformation, and molecular interaction experiments, we will systematically decipher the function and mechanism of this regulatory module. Our work aims to uncover a novel model of MYB-WRKY synergistic regulation. Ultimately, these findings are expected to provide theoretical targets for the molecular improvement of apple aroma traits.

## Results

### *MdAAT2-like* participates in the synthesis of aromatic esters in apple fruit

**Fig. 1:**
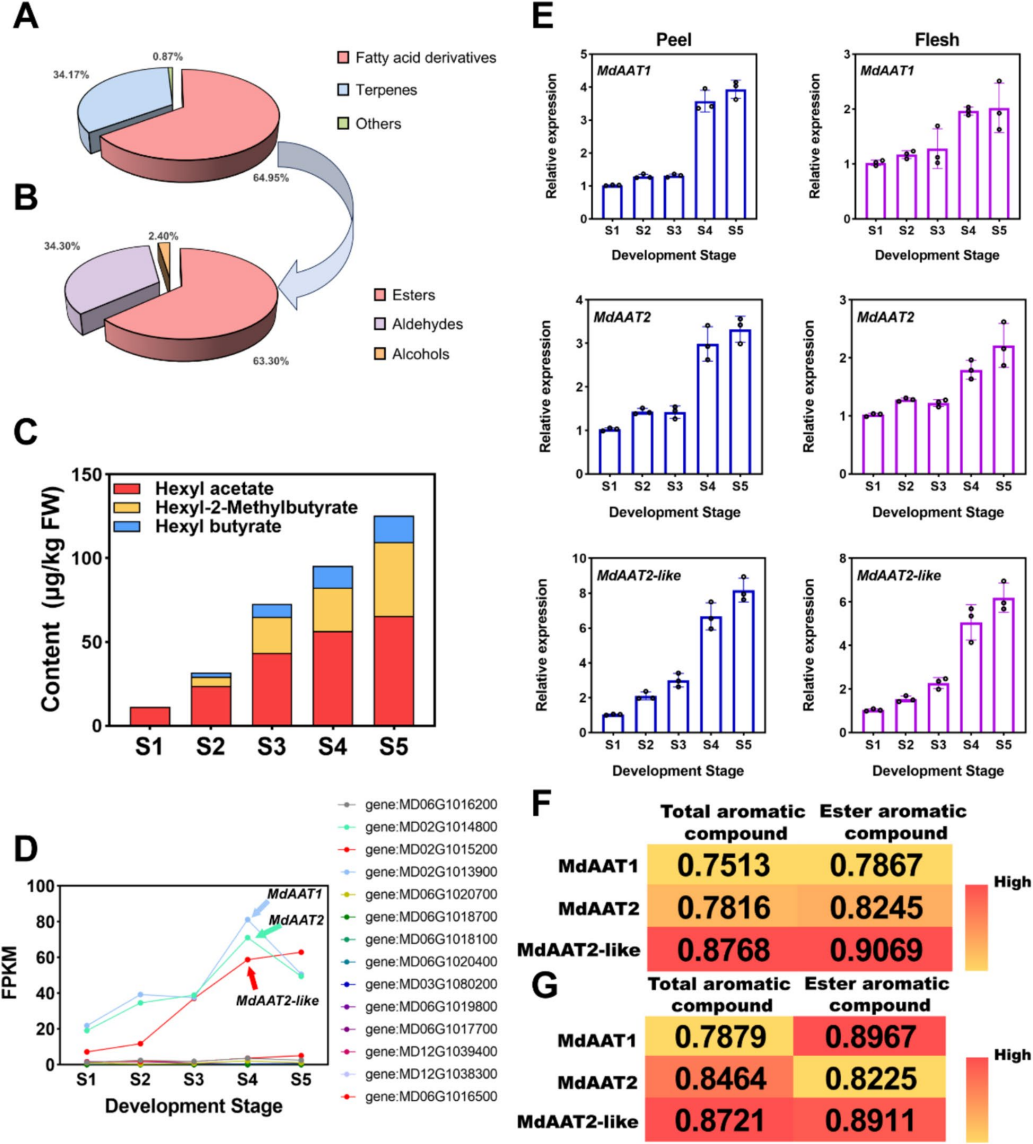
Aromatic ester biosynthesis and MdAAT gene expression during apple development. **A** Percentage of total volatile compounds and (**B**) aromatic esters in ripe apples. **C** Levels of aromatic esters in apple fruits at different developmental stages: S2 (150 DAB), S3 (170 DAB), S4 (180 DAB), S5 (190 DAB), and S6 (200 DAB). **D** Transcript levels of *MdAAT* genes in ripening apples as based on (RNA sequencing) RNA-Seq data. **E** Expression of *MdAAT1*, *MdAAT2* and *MdAAT2-like* in apple fruits as determined by qPCR analysis. **F** Correlation analysis between *MdAAT* gene expression and aromatic compound content in the pericarp. **G** Correlation analysis of *MdAAT* gene expression and aromatic compounds in the fruit flesh. Error bars represent standard error (SE; n = 3)

Aromatic compound accumulation is a key characteristic of mature apple fruits, with lipid-derived volatiles constituting 80% of the total aroma profile. In this study, we found that in ‘Ruixue’ apple fruits at the mature stage (200 days after flowering (DAB)), 64.95% of volatiles originated from the degradation of fatty acids, while 34.17% volatiles were derived from terpenoid compounds (Fig. 1A). Among these fatty acid-derived volatiles, esters accounted for approximately 63.3%, whereas aldehydes and alcohols constituted 34.3% and 2.4%, respectively (Fig. 1B). Consequently, esters represent the most abundant volatiles in mature apple fruits. To further examine changes in the content of aromatic esters during fruit development and ripening, we analyzed apple fruits at five different stages using gas chromatography-mass spectrometry. Our results revealed a significant increase in various aromatic esters, including hexyl acetate, hexyl-2-methylbutyrate, and hexyl butyrate, during fruit ripening (Fig. 1C).

Furthermore, RNA-seq transcriptome analysis of fruit tissues at different developmental stages of ‘Ruixue’ apples identified 14 candidate AAT genes involved in fruit development (Liu et al. 2021) (Table S1). Among them, *MdAAT1*, *MdAAT2*, and *MdAAT2-like* exhibited notably high expression during fruit ripening (Fig. 1D). Quantitative real-time polymerase chain reaction (RT-qPCR) analysis further confirmed that *MdAAT2-like* expression gradually increased in both the peel and flesh as the fruit matured (Fig. 1E). To explore the potential relationship between gene expression and aromatic ester accumulation, we conducted a correlation analysis between the expression levels of these three genes and the total aromatic ester content in ‘Ruixue’ fruits. The results revealed that *MdAAT2-like* exhibited the most significant correlation (*R*^*2*^ = 0.8768) with aromatic ester content (Fig. 1F, G). These findings indicate that MdAAT2-like may play a significant role in contributing to aromatic ester biosynthesis during apple fruit ripening.

**Fig. 2:**
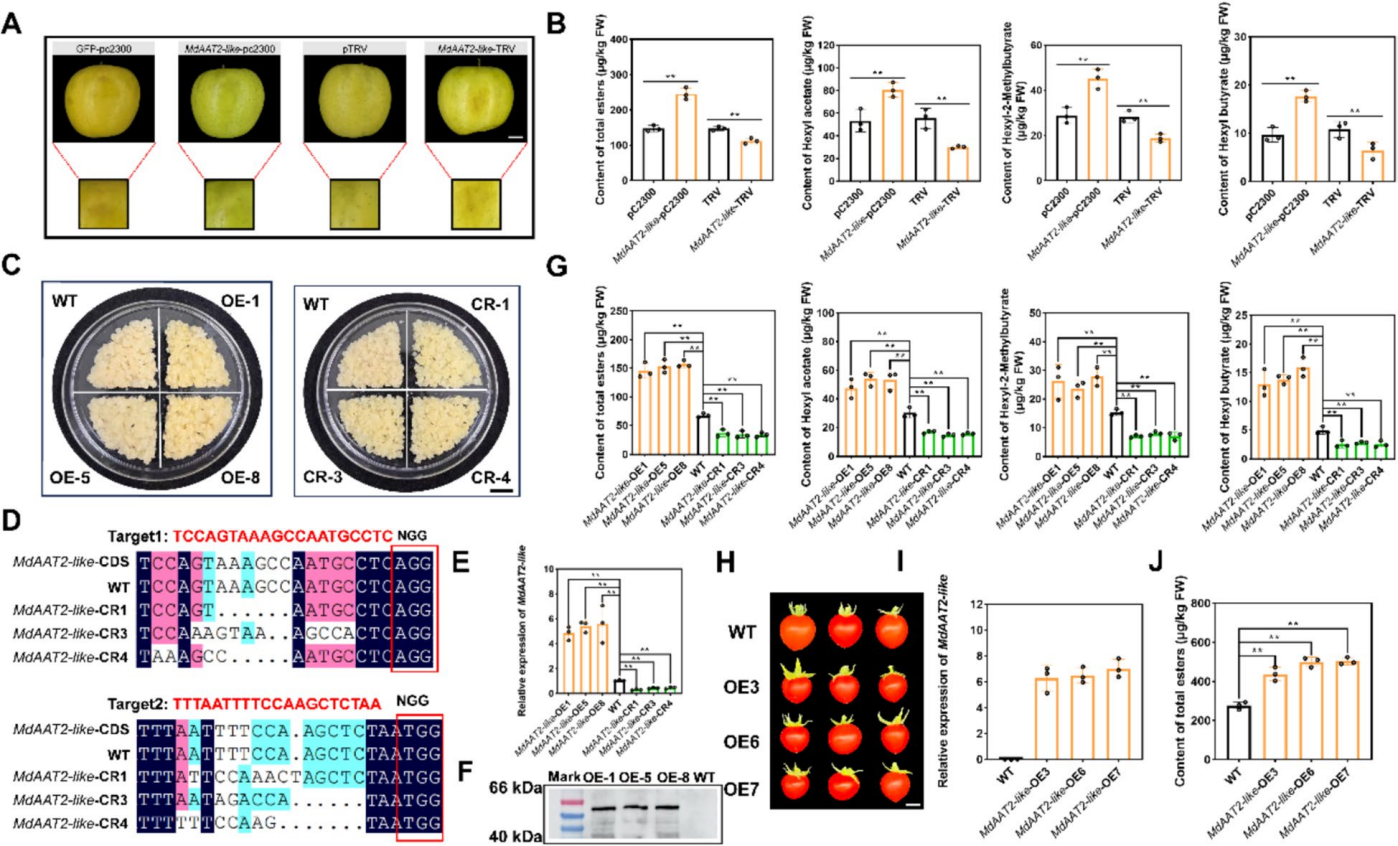
Expression and characterization of *MdAAT2-like* in apple fruit, calli, and tomato. **A** Phenotypic analysis of apple fruits transiently expressing *MdAAT2-like* (scale bar = 1 cm). **B** Content of various aromatic compounds in apple fruit following transient expression of *MdAAT2-like*. **C** Phenotype of *MdAAT2-like* overexpressing ‘Orin’ calli (OE-*MdAAT2-like*−1/5/8) and CRISPR/Cas9 knockout calli (CR-*MdAAT2-like*−1/3/4) (scale bar = 1 cm). **D** Stable knockout construct of *MdAAT2-like*. **E** Relative expression in Orin calli after *MdAAT2-like* overexpression and knockout. **F** Protein accumulation in MdAAT2-like-overexpressing calli by Western blot analysis. **G** Content of various aromatic compounds in calli overexpressing and silencing *MdAAT2-like*. **H** phenotype of MdAAT2-like transgenic tomato (scale bar = 1 cm). **I** Relative expression of *MdAAT2-like* in tomato lines after stable overexpression. **J** Aromatic compounds in *MdAAT2-like*-overexpressing tomato fruits. Data are expressed as mean ± standard error (SE; n = 3); ** indicates P < 0.01, (Student's *t*-test). FW, fresh weight

To further investigate the function of *MdAAT2-like*, we employed transient transformation due to the challenges associated with obtaining genetically modified apple fruits. *Agrobacterium tumefaciens* strains carrying *MdAAT2-like*-pc2300 and *MdAAT2-like*-TRV vectors were introduced into apple fruits under vacuum infiltration (Fig. 2A, Fig. S1). The results showed that fruits overexpressing *MdAAT2-like* had significantly higher aromatic ester content than those in the control group, whereas *MdAAT2-like*-TRV fruits exhibited a marked reduction in aromatic ester content (Fig. 2B). To further elucidate the role of *MdAAT2-like* in aromatic ester biosynthesis, we generated stable transgenic apple calli with *MdAAT2-like* overexpression (OE-1/5/8) and knockout (CR-1/3/4) (Fig. 2C). PCR-based sequencing of *MdAAT2-like* knockout calli revealed multiple substitutions, deletions, and insertions in the target sequence compared to wild-type (WT) calli, confirming successful gene knockout (Fig. 2D). Additionally, RT-qPCR analysis confirmed the successful and stable transformation of *MdAAT2-like* in these calli (Fig. 2E). Consistent with the transient transformation results, *MdAAT2-like* overexpression promoted aromatic ester biosynthesis in transgenic calli, while *MdAAT2-like* knockout resulted in a significant reduction in aromatic ester content (Fig. 2F). To further validate the role of *MdAAT2-like* in aromatic ester biosynthesis, we generated transgenic tomato fruits overexpressing *MdAAT2-like* (Fig. 2G). Compared to WT tomato fruits, transgenic fruits exhibited significantly enhanced aromatic ester content (Fig. 2H). Recombinant MdAAT2-like protein expressed in *E. coli* demonstrated a unique substrate preference profile. While it exhibited significantly lower catalytic efficiency towards canonical aliphatic alcohols (e.g., hexanol) compared to MdAAT1 and MdAAT2, it showed a pronounced preference for medium-chain acyl-CoAs. Specifically, MdAAT2-like displayed superior affinity (lower *K*_*m*_) and catalytic efficiency (higher *K*_*cat*_/*K*_*m*_) for hexanoyl-CoA, providing direct biochemical evidence for its specialized role in shaping the ester volatile profile (Table S2, S3). These findings collectively demonstrate that *MdAAT2-like* plays a crucial role in promoting the synthesis of aromatic esters in fruit.

### MdMYB98-like activates the *MdAAT2-like* promoter to promote aromatic ester biosynthesis in apple fruit

**Fig. 3:**
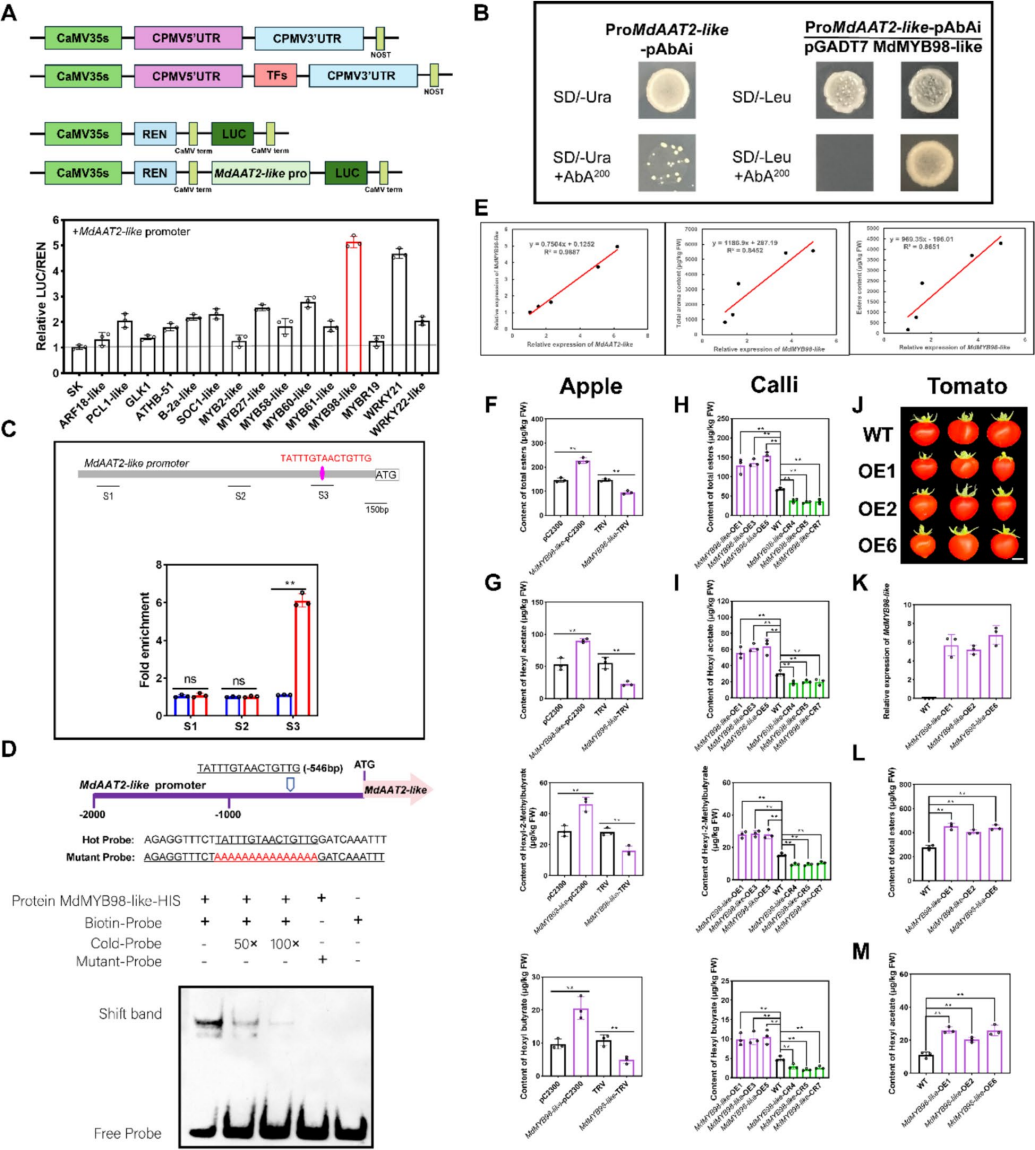
MdMYB98-like promotes aroma synthesis via transcriptional activation of *MdAAT2-like*. **A** Regulation of the *MdAAT2-like* promoter by transcription factors. Means and standard errors were calculated from three replicates. **B** Y1H assay demonstrating MdMYB98-like binding to the *MdAAT2-like* promoter. SD-Ura medium containing Aureobasidin A (AbA) was used to test promoter self-activation. AD-empty and pAbAi-*MdAAT2-like* served as negative controls. **C** ChIP-qPCR analysis of MdMYB98-like binding to the *MdAAT2-like* promoter in vivo. Three regions (S1–S3) on the *MdAAT2-like* promoter were analyzed, with calli containing GFP sequences used as a negative control. Data represent mean ± standard error (SE; N = 3). **D** EMSA showing MdMYB98-like binding to the *MdAAT2-like* promoter. Biotin-labeled dsDNA probes were used. Red letters indicate mutant probes; + or—indicate the presence or absence of a specific probe. **E** Correlation between MdMYB98-like and *MdAAT2-like* expression levels and the content of various aromatic compounds. **F-G** Effect of transient expression of *MdMYB98-like* on the content of aromatic compounds in apple fruit. **H-I** Effects of *MdMYB98-like* overexpression and knockout on aromatic compound content in ‘Orin’ calli. **J-M** *MdMYB98-like*-overexpressing tomato fruits show higher aromatic compound contents than WT fruits (scale bar = 1 cm). Error bars indicate mean ± standard error (SE; n = 3), ** indicates significant differences at P < 0.01 (Student's *t*-test). All data were processed using the described statistical methods. FW, Fresh weight

Bioinformatic analysis of the *MdAAT2-like* promoter sequence predicted binding sites for multiple transcription factor families, including ERF, NAC, MYB, MADS, bHLH, WRKY, and bZIP (Fig. S2). To identify the transcription factors binding to these sites, a yeast one-hybrid (Y1H) screen was performed, which yielded 15 candidate transcription factors. A dual-luciferase reporter (Hellens et al. 2005) assay in *Nicotiana benthamiana* leaves was employed to evaluate their regulatory effects on the *MdAAT2-like* promoter. The results demonstrated that MdMYB98-like and MdWRKY21 significantly activated *MdAAT2-like* expression (Fig. 3A; Table S4). To further investigate MdMYB98-like, a key regulator of aromatic ester biosynthesis, we conducted a Y1H assay, which confirmed its interaction with the *MdAAT2-like* promoter (Fig. 3B). To confirm this interaction in vivo, a chromatin immunoprecipitation (ChIP)-qPCR assay was performed. The results showed significant enrichment of the *MdAAT2-like* promoter fragment in MdMYB98-like-expressing apple calli, demonstrating that MdMYB98-like binds to the *MdAAT2-like* promoter in vivo (Fig. 3C). EMSAs further verified the specific binding of MdMYB98-like to the *MdAAT2-like* promoter, with the binding completely abolished when the predicted binding site was mutated. Additionally, competitive cold probes progressively weakened the binding strength in a concentration-dependent manner (Fig. 3D). Subsequently, we analyzed the expression level of *MdMYB98-like* during fruit development and observed a progressive increase in its expression level as the fruit matured. This trend was consistent with the accumulation patterns of total aromatic compounds and aromatic esters throughout fruit development (Fig. S3). To further explore potential regulatory relationships, we conducted a correlation analysis between *MdMYB98-like* expression, *MdAAT2-like* expression, and the levels of total aromatic compounds and aromatic esters. Our findings revealed a significant positive correlation of *MdMYB98-like* expression with *MdAAT2-like* expression, as well as with total aromatic compound and aromatic ester accumulation (Fig. 3E). This suggests that MdMYB98-like may positively regulate the transcriptional activity of *MdAAT2-like*, thereby promoting the accumulation of aromatic esters in fruits.

To investigate the regulatory role of *MdMYB98-like* in *MdAAT2-like* expression and aromatic ester accumulation, we overexpressed and silenced *MdMYB98-like* in apple fruit. RT-qPCR and Western blot analyses of *MdMYB98-like* transgenic fruit revealed significantly elevated *MdMYB98-like* expression in overexpressed fruits compared to the control group (Fig. S4A, B). Concurrently, *MdAAT2-like* expression and aromatic ester content were significantly increased (Fig. S4C; Fig. 3F, G). In contrast, silencing *MdMYB98-like* resulted in nearly undetectable transcript levels, leading to a significant reduction in both *MdAAT2-like* expression and aromatic ester content in apple fruit. To further elucidate the function of *MdMYB98-like*, we generated *MdMYB98-like* overexpression and knockout calli to assess their impact on aromatic ester biosynthesis (Fig. S5; Fig. S6A, B). Overexpression of *MdAAT2-like* significantly upregulated *MdAAT2-like* expression and increased aromatic ester content compared to that observed in WT calli. Conversely, *MdMYB98-like* knockout led to a marked downregulation of *MdAAT2-like* expression and decrease in aromatic ester content (Fig. 3H, I; Fig. S6C). To validate these findings in a heterologous system, we overexpressed *MdMYB98-like* in tomato plants (Fig. 3J). The results showed a significant upregulation of *MdMYB98-like* expression in transgenic tomato lines compared to those in WT tomatoes (Fig. 3K). Upon fruit ripening, the aromatic ester content in overexpressed tomato fruits was significantly higher than that in WT controls (Fig. 3L, M). These findings demonstrate that *MdMYB98-like* positively regulates *MdAAT2-like* expression and plays a crucial role in the biosynthesis of aromatic esters in apple fruit.

**Fig. 4:**
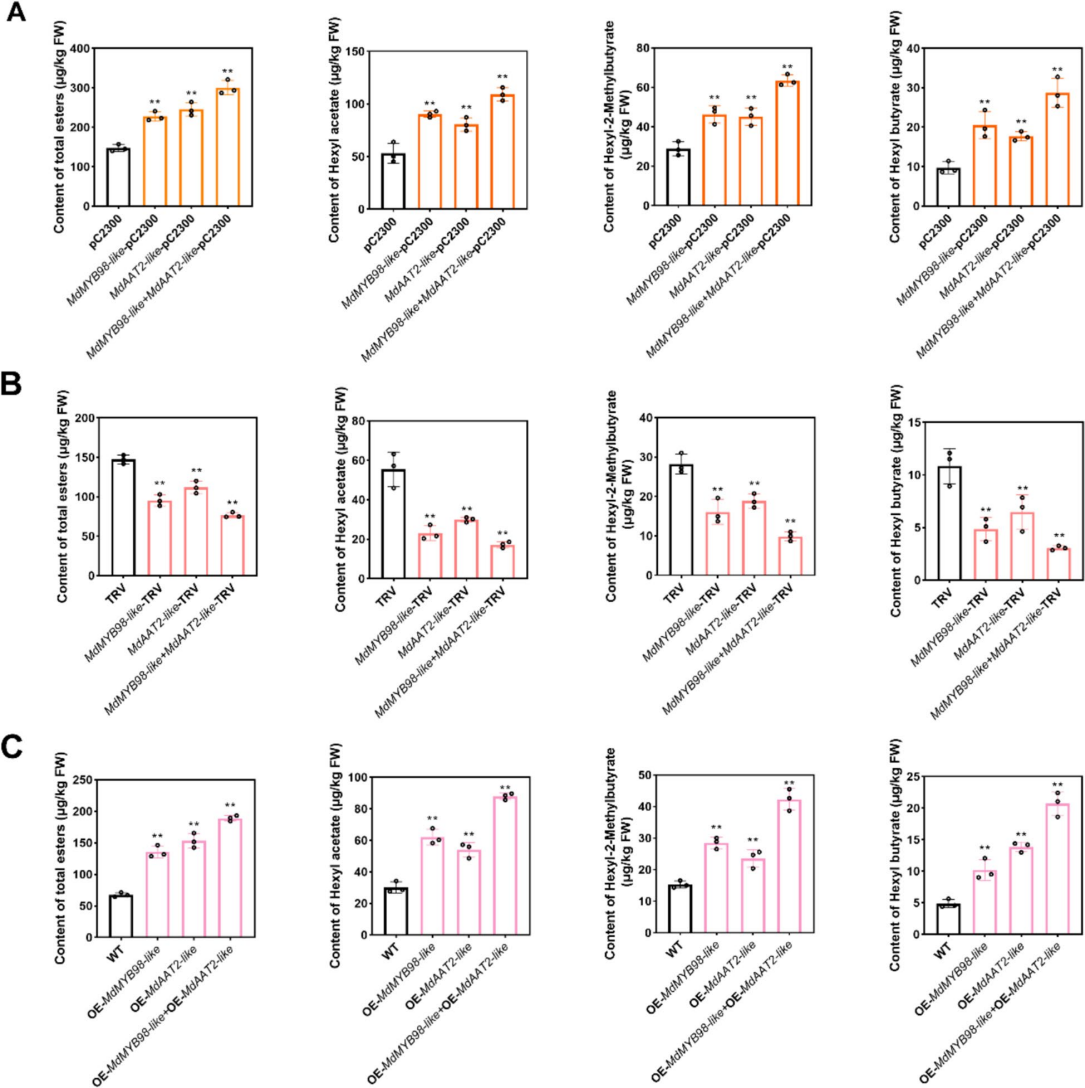
Changes in aroma content following simultaneous overexpression of *MdMYB98-like* and *MdAAT2-like*. **A** Aromatic ester contents in apple fruit overexpressing *MdMYB98-like*, *MdAAT2-like*, or both (*MdMYB98-like* + *MdAAT2-like*). **B** Aromatic ester content in apple fruit silencing *MdMYB98-like*, *MdAAT2-like*, and both (*MdMYB98-like* + *MdAAT2-like*). **C** Aromatic compound content in apple calli overexpressing *MdMYB98-like*, *MdAAT2-like*, and both. Error bars indicate mean ± standard error (SE; n = 3), ** indicates significant differences at P < 0.01 (Student's *t*-test). FW, Fresh weight

To further investigate the roles of *MdMYB98-like* and *MdAAT2-like* in the synthesis of aromatic esters in apples, we overexpressed both genes in apple fruit (Fig. S7A-C). The results showed that simultaneous overexpression of *MdMYB98-like* and *MdAAT2-like* led to a significantly higher aromatic ester content compared to that in the control group and fruits overexpressing either gene individually (Fig. 4A). Conversely, silencing both *MdMYB98-like* and *MdAAT2-like* resulted in a significant reduction in aromatic ester content, which was significantly lower than in the control and fruits with individual gene silencing (Fig. 4B). To further validate these findings, we generated stable transgenic apple calli co-overexpressing *MdMYB98-like* and *MdAAT2-like* (Fig. S7D-F). Quantification of aromatic esters in these calli revealed a marked increase in ester content when both genes were overexpressed simultaneously (Fig. 4C). These findings suggest that *MdMYB98-like* and *MdAAT2-like* function synergistically to enhance the synthesis of aromatic esters in apple fruit, with their combined presence exerting a stronger regulatory effect than their individual expression.

### MdWRKY21 promotes the synthesis of aromatic esters in apple fruit

**Fig. 5:**
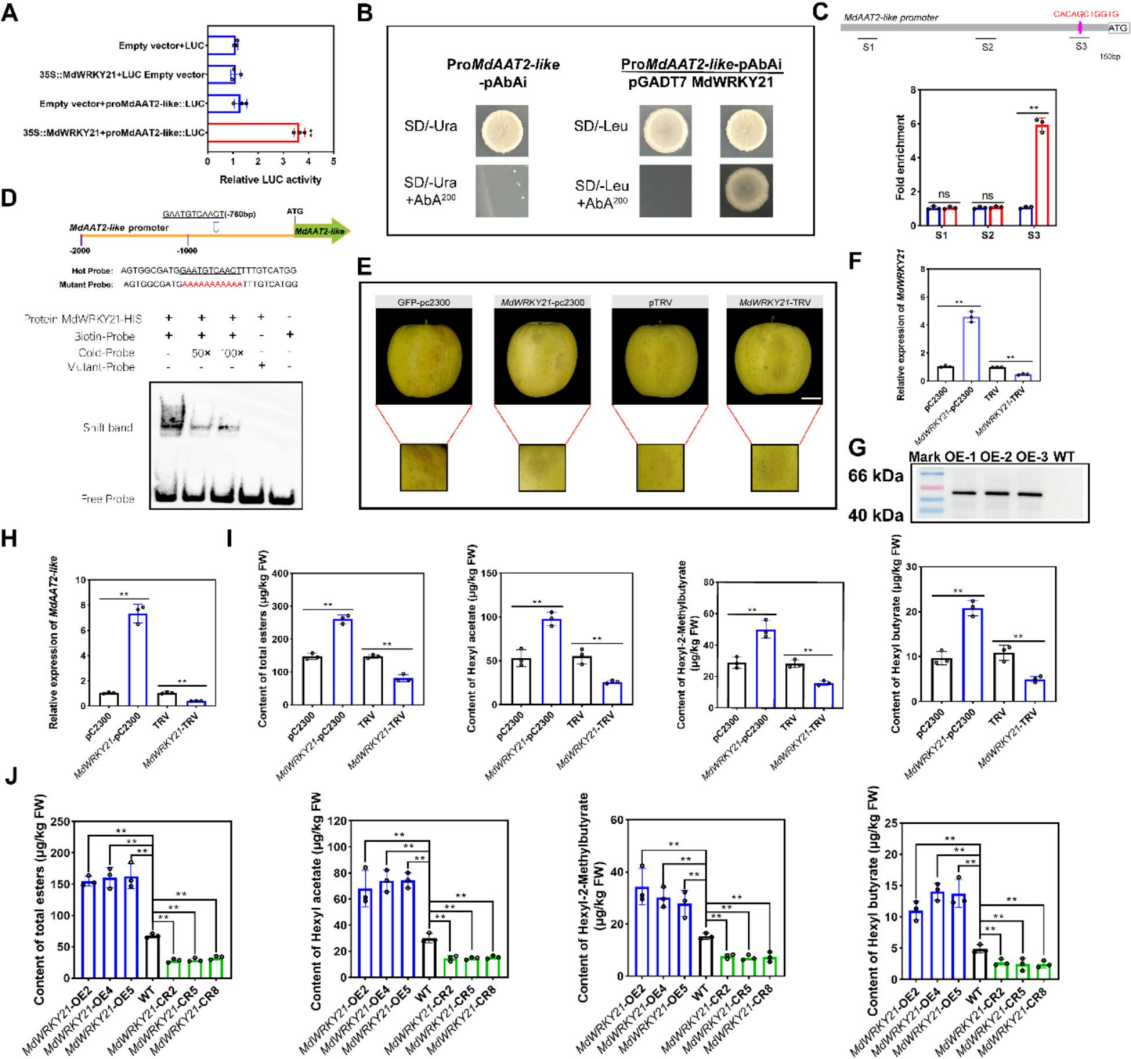
MdWRKY21 upregulates *MdAAT2-like* to enhance aromatic compound biosynthesis. **A** Luciferase activity assay showing that MdWRKY21 activates *MdAAT2-like* promoter activity. **B** Y1H assay confirming MdWRKY21 binding to the *MdAAT2-like* promoter. **C** EMSA verifying MdWRKY21 binding to the *MdAAT2-like* promoter. **D** Chromatin immunoprecipitation (ChIP-qPCR) confirming in vivo binding of MdWRKY21 to the *MdAAT2-like* promoter. **E** Phenotypes of apple transiently expressing *MdWRKY21*. Bars = 1 cm. Relative expression of *MdWRKY21* (**F**) and protein level of MdWRKY21 (**G**) after transient *MdWRKY21* overexpression. **H** Relative expression of *MdAAT2-like* after transient *MdWRKY21* overexpression. **I** Contents of aromatic compounds after transient expression of *MdWRKY21*. **J** Content of aromatic compounds in 'Orin' calli overexpressing and knock-out *MdWRKY21*. The results from three separate biological replicates are expressed as mean ± standard error (SE). Asterisks indicate significant differences compared to the control (**P < 0.01) based on a Student’s *t*-test. ns indicates ‘not significant’ (Student’s *t*-test)

To determine whether MdWRKY21 directly regulates *MdAAT2-like*, we first performed luciferase (LUC) and Y1H assays. The results confirmed that MdWRKY21 directly binds to the *MdAAT2-like* promoter (Fig. 5A, B). Furthermore, a ChIP-qPCR assay confirmed that MdWRKY21 directly binds to the *MdAAT2-like* promoter in vivo, further supporting its regulatory role in aromatic ester biosynthesis in apples (Fig. 5C). This interaction was further validated using an EMSA, which showed that mutating the predicted binding site eliminated the binding. Additionally, increasing the concentration of cold probe competitors led to a gradual reduction in binding affinity (Fig. 5D). To further elucidate the relationship between MdWRKY21 and MdAAT2-like, we conducted a correlation analysis assessing the expression levels of *MdWRKY21*, *MdAAT2-like*, and *MdMYB98-like*, alongside total aromatic compound content and aromatic ester content. The results revealed a significant positive correlation between *MdWRKY21* and all these factors, further supporting the hypothesis that MdWRKY21 enhances the transcriptional activity of *MdAAT2-like* (Fig. S8).

To investigate the role of *MdWRKY21* in the synthesis of aromatic esters in apple fruit, we conducted overexpression and silencing experiments (Fig. 5E-J). The results indicated that *MdWRKY21* overexpression significantly increased ester content in apple fruit compared to that observed in the WT control. Conversely, the suppression of *MdWRKY21* reduced both the transcript levels of *MdAAT2-like* and ester content (Fig. 5H, I). To further examine *MdWRKY21* function, we established *MdWRKY21* overexpression and knockout lines in ‘Orin’ calli (Fig. S9A-C) and measured the expression levels of *MdAAT2-like*, along with their aromatic ester content. In *MdWRKY21*-overexpressing calli, both *MdAAT2-like* expression and aromatic ester content were significantly elevated compared to those of the WT calli (Fig. 5I; Fig. S9D). In contrast, knockout of *MdWRKY21* resulted in a significant reduction in *MdAAT2-like* expression and aromatic ester accumulation (Fig. 5I; Fig. S9D). Furthermore, overexpression of *MdWRKY21* in transgenic tomatoes led to significantly higher expression levels of *MdWRKY21* compared to WT tomatoes (Fig. S10A, B). Additionally, the content of ester substances was markedly increased in the overexpression lines (Fig. S10C, D). These findings underscore the pivotal role of *MdWRKY21* in regulating *MdAAT2-like* expression and enhancing aromatic ester biosynthesis in fruit.

Additionally, we performed simultaneous overexpression and silencing of *MdWRKY21* and *MdAAT2-like* in apple fruits (Fig. S11A-C). Fruits co-overexpressing both genes exhibited significantly higher aromatic ester content than those overexpressing either gene individually (Fig. S12A). Similarly, silencing both genes resulted in a significant reduction in ester content than silencing either gene alone (Fig. S12B). Furthermore, we generated stable co-overexpression lines of *MdWRKY21* and *MdAAT2-like* in apple calli (Fig. S11D, E). Quantitative analysis of aromatic ester content in these transgenic calli demonstrated a significant increase in aromatic ester levels when both genes were overexpressed simultaneously (Fig. S12C). These results indicate that *MdWRKY21* enhances the synthesis of aromatic esters in apple fruit, and its regulatory effect is more pronounced when acting together with *MdAAT2-like*.

### MdMYB98-like and MdWRKY21 promote their expression by interacting with corresponding promoters

**Fig. 6:**
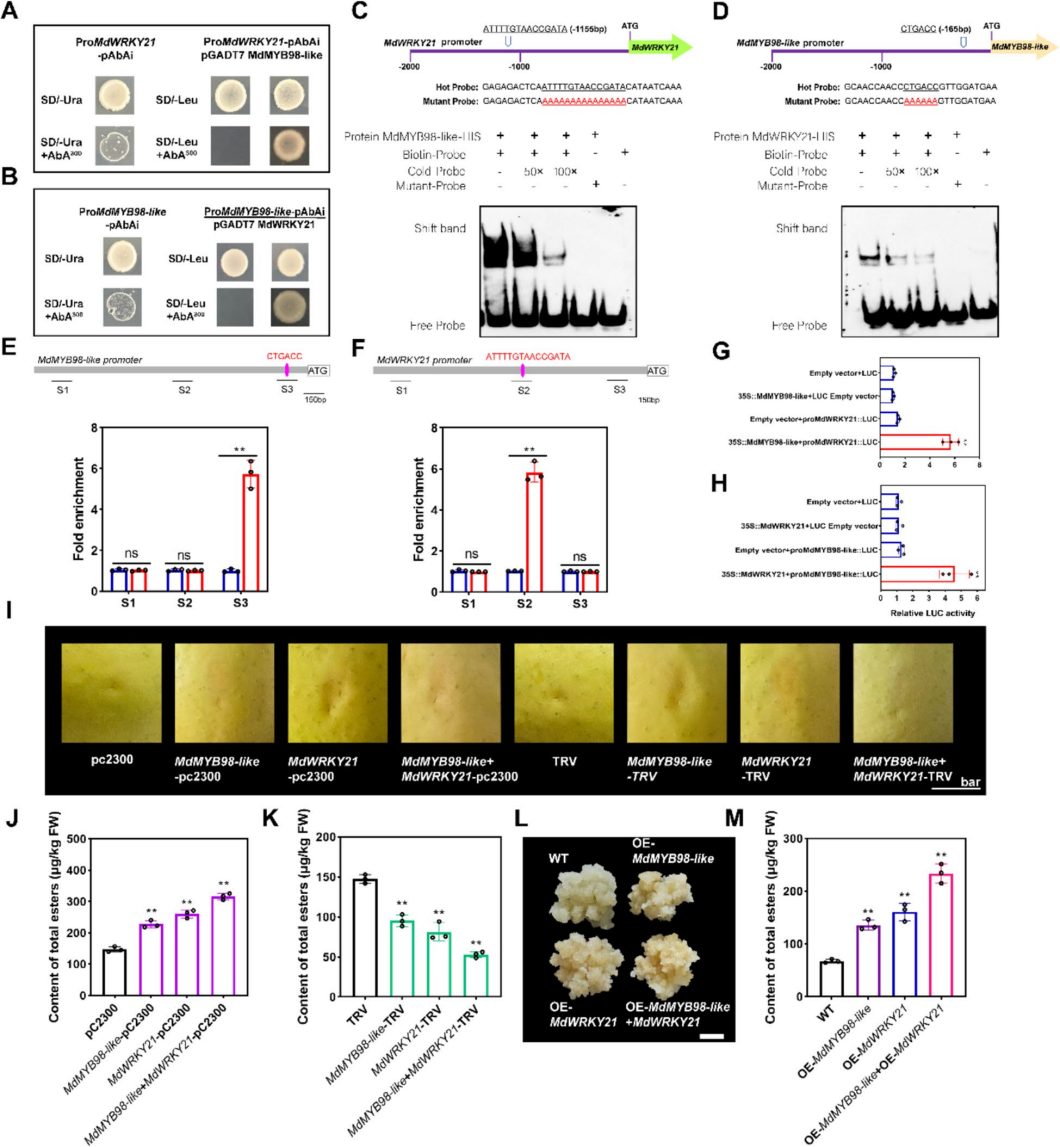
A positive feedback loop between MdMYB98-like and MdWRKY21 boosts volatile ester formation. **A**, **B** Y1H assays revealed mutual promoter binding between MdMYB98-like and MdWRKY21 (300 mM AbA). **C**, **D** EMSA analysis reveals bidirectional binding interactions: MdMYB98-like-HIS binds to the MdWRKY21 promoter, while MdWRKY21 interacts with the motif in the MdMYB98-like promoter. Red letters indicate variant probes; + or—indicates the presence or absence of a particular probe. **E**, **F** ChIP-qPCR analysis shows mutual in vivo binding of MdMYB98-like to the MdWRKY21 promoter and MdWRKY21 to the MdMYB98-like promoter, with three promoter regions (S1–S3) analyzed using GFP-containing calli as negative controls; data are presented as mean ± standard error (SE; n = 3). **G**, **H** LUC assay confirms reciprocal in vivo binding between MdMYB98-like and MdWRKY21 to their respective promoters. **I** Phenotypes of apples after transient *MdMYB98-like* and *MdWRKY21* infection. Scale bar = 2 cm. **J** Ester content in apple fruit overexpressing *MdMYB98-like*, *MdWRKY21* and co-expressing *MdMYB98-like* + *MdWRKY21*. **K** Content of ester in fruits silencing *MdMYB98-like*, *MdWRKY21* and co-silencing *MdMYB98-like* + *MdWRKY21*. **L** Phenotype of *MdMYB98-like* and *MdWRKY21* overexpressing ‘Orin’ calli. Bars = 1 cm. **M** Ester content in 'Orin' calli overexpressing *MdMYB98-like*, *MdWRKY21* and co-expressing *MdMYB98-like* + *MdWRKY21*. Scale bar = 2 cm. Error bars indicate mean ± SE (n = 3), and ** indicates a significant difference at *P* < 0.01 (Student’s *t*-test); ns indicates Not Significant (Student’s *t*-test). FW, fresh weight

We observed that *MdWRKY21* expression levels in apple fruit and calli plants were altered following the transformation of transgenic *MdMYB98-like*. Similarly, transformation of *MdWRKY21* led to changes in *MdMYB98-like* expression (Fig. S13). These findings suggest a potential regulatory relationship between *MdMYB98-like* and *MdWRKY21*. To validate this hypothesis, we conducted Y1H assays, which confirmed that MdMYB98-like and MdWRKY21 can bind to each other's promoter regions (Fig. 6A, B). EMSAs further demonstrated that MdMYB98-like interacts with the cis-acting element in the *MdWRKY21* promoter, while MdWRKY21 binds to the W-box in the *MdMYB98-like* promoter (Fig. 6C, D). ChIP-qPCR results revealed that *MdMYB98-like* overexpressing (OE-*MdMYB98-like*) calli exhibited greater enrichment of *MdWRKY21* promoter fragments compared to controls (Fig. 6E). Similarly, OE-*MdWRKY21* calli showed an increased abundance of OE-*MdMYB98-like* promoter fragments (Fig. 6F). These findings confirm that MdMYB98-like and MdWRKY21 bind to their corresponding promoters both in vitro and in vivo. Additionally, fluorescence assays in tobacco leaves showed that co-expression of 35S::MdMYB98-like with pro*MdWRKY21*::LUC resulted in significantly higher fluorescence intensity compared to the expression of pro*MdWRKY21*::LUC alone (Fig. 6G). Similarly, co-expression of 35S::MdWRKY21 with pro*MdMYB98-like*::LUC led to significantly greater fluorescence intensity than the expression of pro*MdMYB98-like*::LUC alone (Fig. 6H). These findings demonstrate that MdMYB98-like and MdWRKY21 positively regulate the activity of their corresponding promoters. Furthermore, apple fruits co-overexpressing *MdMYB98-like* and *MdWRKY21* exhibited significantly higher aromatic ester content compared to those overexpressing either gene alone (Fig. 6I-M; Fig. S14).

### MdMYB98-like interacts with MdWRKY21 protein

**Fig. 7:**
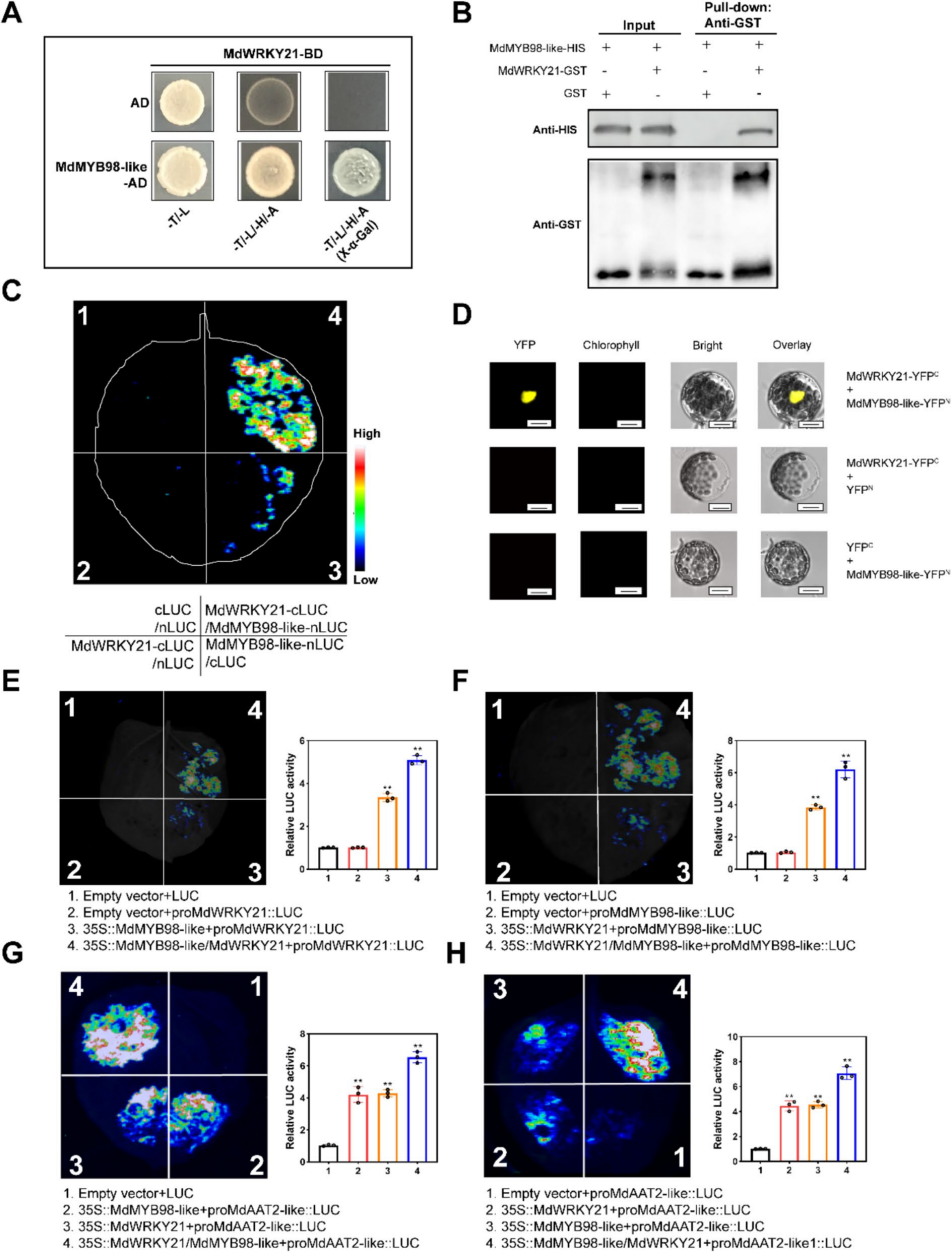
MdWRKY21 interacts with MdMYB98-like to promote transcriptional activation of the MdAAT2-like promoter. **A** Y2H assay showing the interaction of MdMYB98-like with MdWRKY21. **B** Pull-down assay proves that MdMYB98-like interacts with MdWRKY21. The “ + ” and “ − ” indicate the presence and absence of the indicated protein, respectively. **C** In LCI assay MdMYB98-like interacts with MdWRKY21. **D** BiFC assay shows that MdMYB98-like interacts with MdWRKY21. LUC activity analysis reveals that the interaction between MdWRKY21 and MdMYB98-like enhances the transcriptional activation of the MdMYB98-like to the *MdWRKY21* promoter (**E**) as well as the transcriptional activation of MdWRKY21 to *MdMYB98-like* (**F**). **G**, **H** LUC activity analysis confirms that the interaction between MdWRKY21 and MdMYB98-like enhances each other's transcriptional activation of the *MdAAT2-like* promoter. Error bars indicate mean ± standard error (SE) (n = 3), ** indicates significant difference, P < 0.01 (Student's *t*-test); ns indicates insignificant difference (Student's *t*-test)

In plant metabolism, MYB and WRKY transcription factors often interact to regulate gene expression. Given that MdMYB98-like and MdWRKY21 belong to the MYB and WRKY transcription factor families, respectively, we hypothesized that they may physically interact. To test this, we performed a yeast two-hybrid (Y2H) assay, which confirmed the interaction between MdMYB98-like and MdWRKY21 (Fig. 7A). This interaction was further validated through a pull-down assay, where MdMYB98-like-HIS successfully pulled down MdWRKY21-GST, demonstrating their direct binding in vitro (Fig. 7B). The luciferase complementation imaging (LCI) experiment demonstrated that tobacco leaves co-expressing MdMYB98-like-nLUC and MdWRKY21-cLUC exhibited strong fluorescence signals, providing additional evidence of their interaction in vivo (Fig. 7C). Additionally, a BiFC assay revealed stable yellow fluorescent protein (YFP) signals in protoplasts coexpressing MdMYB98-like-YFP^N^ and MdWRKY21^C^, confirming their interaction in vivo (Fig. 7D).

To investigate whether this protein interaction influences the transcriptional activation of each other's promoters, we conducted LUC reporter assay. The result demonstrated that in the presence of both MdMYB98-like and MdWRKY21, the activity of the *MdWRKY21* promoter was significantly higher than when MdMYB98-like was present alone (Fig. 7E). Similarly, MdWRKY21 significantly enhanced the activation of the *MdMYB98-like* promoter, and when both proteins were co-expressed, *MdMYB98-like* promoter activity was significantly higher than when *MdWRKY21* was expressed alone (Fig. 7F). These findings demonstrate that the interaction between MdMYB98-like and MdWRKY21 significantly enhances the transcriptional activation of each other’s promoters.

### MdMYB98-like interacts with MdWRKY21 to promote the transcriptional activation of the *MdAAT2-like* promoter

Experimental results indicate that both MdMYB98-like and MdWRKY21 bind to the *MdAAT2-like* promoter and enhances its expression. To further determine whether their interaction influences this regulatory effect, we conducted LUC reporter assay. The results demonstrated that the simultaneous presence of MdMYB98-like and MdWRKY21 significantly enhanced *MdAAT2-like* promoter activity compared to either transcription factor alone (Fig. 7G, H).

## Discussion

Aromatic esters serve as the principal components responsible for the characteristic aroma profile of apple fruits, with their composition and concentration directly determining the fruit's flavor quality (Espino-Díaz et al. 2016). As members of the BAHD acyltransferase family, AATs catalyze the esterification reactions between various acyl-CoAs and alcohol substrates, playing a pivotal role in generating ester diversity (Zhu et al. 2022). While *MdAAT1* and *MdAAT2* have been established as key genes catalyzing ester biosynthesis in apple (Souleyre et al. 2005; Li et al. 2006a, b), whether functional diversification exists among other family members and whether the regulatory network is more complex remains to be thoroughly investigated.

Building upon the confirmed expression of *MdAAT1* and *MdAAT2*, our study further identifies *MdAAT2-like* as a novel key member in ester biosynthesis. In contrast to *MdAAT1*/*MdAAT2*, which primarily prefer short-chain aliphatic alcohols (Souleyre et al. 2005), our in vitro enzymatic analyses revealed that MdAAT2-like exhibits significantly higher catalytic efficiency and affinity toward medium-chain acyl-CoAs (e.g., hexanoyl-CoA). This unique acyl-donor preference distinguishes its function from the classical MdAAT1/2 enzymes, thereby defining a unique functional role for MdAAT2-like and broadening our understanding of functional diversity among apple ester synthases.

Several MYB transcription factors, including *MdMYB1*, *MdMYB6*, and *MdMYB85*, have been previously reported to regulate *MdAAT2* and *MdAAT1* expression, respectively (Li et al. 2014, 2023). In this study, we demonstrate that MdMYB98-like acts as a direct upstream regulator of *MdAAT2-like*. More significantly, we discovered that MdMYB98-like interacts with a WRKY family transcription factor, MdWRKY21, forming a complex that synergistically enhances the transcriptional activation of *MdAAT2-like*. To investigate the regulatory specificity of this transcriptional module, we further compared its effects on the homologous genes *MdAAT1* and *MdAAT2*. The results indicated that although the expression of *MdAAT1* and *MdAAT2* was modulated to some extent, their response amplitudes were significantly weaker than that of *MdAAT2-like* (Fig. S15). This suggests that the MdMYB98-like–MdWRKY21 complex exhibits preferential regulation within this AAT gene family, with *MdAAT2-like* serving as its primary target. This finding further solidifies the important role of *MdAAT2-like* within this regulatory network, thereby opening new avenues for future investigations into the potential cooperative relationships among MdAAT family members in shaping ester diversity.

Although WRKY transcription factors have been implicated in processes like apple fruit softening and acid metabolism (Wang et al. 2023b, 2024), their direct regulatory function in aromatic ester biosynthesis had not been established. Our findings thus define a previously unrecognized role for MdWRKY21 in this pathway, establishing WRKY factors as functional components of the apple aroma regulatory network and contributing to a more comprehensive understanding of its complexity. Although MYB-WRKY interactions have precedents in plant secondary metabolism (Zhang et al., 2024c), their functional role in fruit aroma biosynthesis had, to our knowledge, not been established. Our work elucidates the mechanism of such an interaction in the synthesis of aromatic esters. Furthermore, we discovered a positive feedback transcriptional circuit between MdMYB98-like and MdWRKY21. This sophisticated regulatory design may underline the fast accumulation of aromatic compounds during fruit ripening, providing a novel mechanistic perspective on how plants precisely regulate the synthesis of volatile metabolites.

**Fig. 8:**
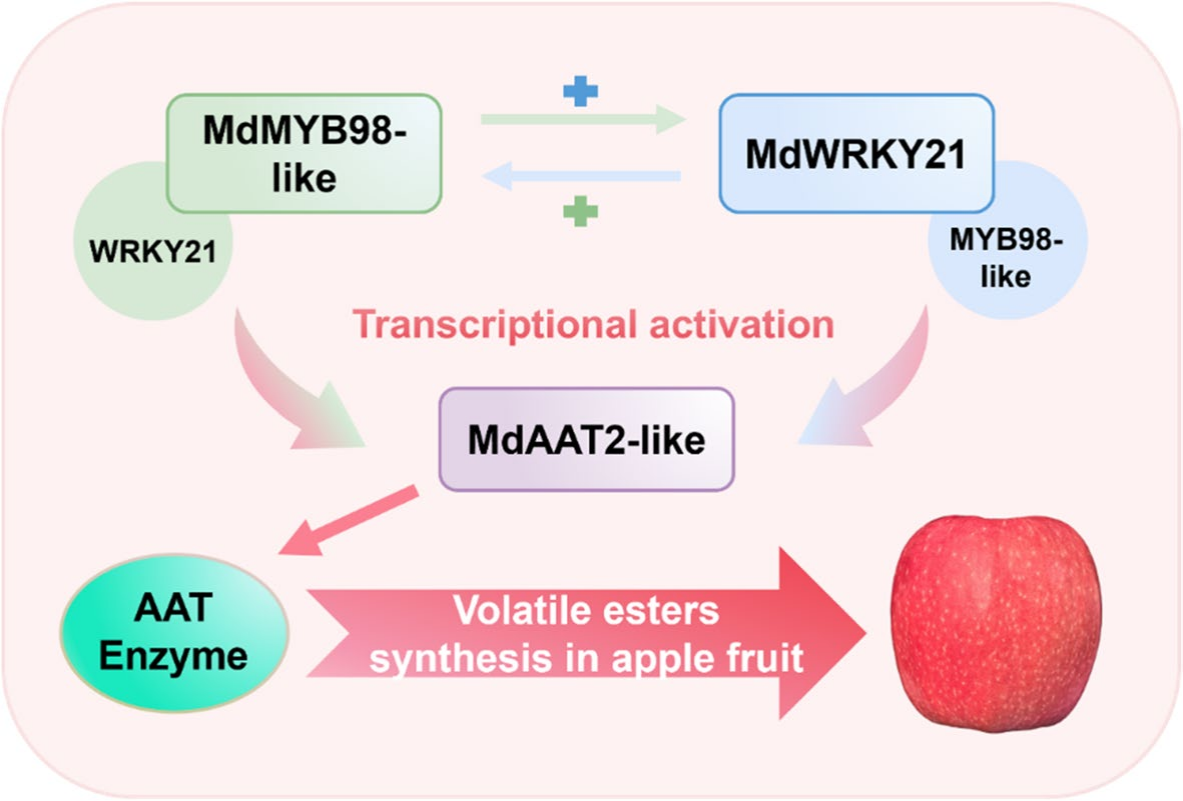
Proposed model for the fine-tuning of aromatic ester biosynthesis in apple fruit regulated by the MdMYB98-like-MdWRKY21 transcription module

In summary, we propose a regulatory module centered on the MdMYB98-like–MdWRKY21 transcriptional complex, which employs a positive feedback loop for self-amplification and precisely drives *MdAAT2-like* expression to promotor ester synthesis (Fig. 8). This model not only deepens the understanding of aroma biosynthesis in apple but also provides new theoretical targets for improving fruit flavor quality through multi-gene coordinated regulation.

MdMYB98-like and MdWRKY21 transcription factors constitute a regulatory module that fine-tunes aromatic ester biosynthesis in apple fruit. These transcription factors not only activate each other's expression, forming a positive feedback loop that amplifies the regulatory signal, but also interact to form a protein complex. This MYB-WRKY complex synergistically activates the promoter of *MdAAT2-like*.

## Material and methods

### Plant materials

‘Ruixue’ apples, obtained from the Baishui Apple Experimental Station at Northwest A&F University, Shaanxi Province, China, were harvested 200 DAB, selecting those with uniform size and maturity and excluding those with physical damage or defects. For transient expression analysis, ‘Golden Crown’ apples were also harvested at 200 DAB.

‘Orin’ calli, used for genetic transformation, were maintained under dark conditions at 24 °C on solid Murashige and Skoog (MS) medium supplemented with 0.8 mg·L^−1^ 6-benzylaminopurine (6-BA) and 1.5 mg·L^−1^ 2,4-dichlorophenoxyacetic acid (2,4-D). Subculturing was performed every 15 d to ensure stable growth. *N. benthamiana* plants used in this study were cultivated under controlled conditions as previously described (Wang et al. 2023a). Additionally, ‘Micro-Tom’ tomato (*Solanum lycopersicum*) plants were cultivated at 24 °C under a 16-h light/8-h dark photoperiod.

### Determination of volatile contents

Headspace solid-phase microextraction (HS-SPME) was performed following a previously described method to identify aromatic esters present in apple fruits (Liu et al. 2023). Each sample consisted of 5 g of apple tissue, sourced from near the skin, which was mixed with 10 µL of a 0.4 mg·mL^−1^ solution of 3-Nonanone, which served as an internal standard. At a temperature of 45 °C, volatile compounds were extracted using a fiber-coated needle with 50/30 µm divinylbenzene/carboxen/polydimethylsiloxane (DVB/CAR/PDMS) SPME fibers (Supelco, Bellefonte, PA, USA). To enhance data reliability, every experiment was conducted with three biological replicates.

### RNA extraction and RT-qPCR analysis

RNA extraction was conducted using apple calli, apple fruits, and tomato fruits employing an RNA extraction kit from Tiangen Biotech Co., Ltd, in accordance with the manufacturer's instructions. The resultant purified RNA was subsequently reverse-transcribed into complementary DNA (cDNA) and analyzed using RT-qPCR on a LightCycler 96 system (Roche, Switzerland). For the quantification of gene expression levels, the 2⁻^ΔΔCt^ method was employed (Livak & Schmittgen 2001), utilizing *MdActin* as the normalization reference gene. RT-qPCR assays were performed on a Bio-Rad iCycler iQ5 system (Hercules, CA, USA), and all primers were synthesized by Sangon Biotech located in Shanghai, China (refer to Table S4). Each sample underwent triplicate analysis to ensure reliable results.

### Stable transformation of apple calli and tomato

The coding sequences (CDSs) of *MdAAT2-like*, *MdMYB98-like*, and *MdWRKY21* were all cloned into the pCAMBIA 2300 expression vector, which carries a kanamycin resistance marker, to facilitate stable overexpression. For targeted gene knockout, CRISPR/Cas9 target sites were identified using an online tool (http://crispr.hzau.edu.cn/CRISPR2/), and corresponding primers were designed. The single guide RNA (sgRNA) sequences were subsequently inserted into the pHSE401 vector. The recombinant plasmids were then introduced into *A. tumefaciens* strain EHA105 for genetic transformation. For apple calli transformation, the recombinant *Agrobacterium* strains harboring pCAMBIA2300 or pHSE401 constructs were used to infiltrate ‘Orin’ apple calli following standard protocols. Additionally, pCambia2300-*MdAAT2-like*, pCambia2300-*MdMYB98-like*, and pCambia2300-*MdWRKY21* constructs were transformed into ‘Micro-Tom’ tomato, according to a previously described method (Zhang et al. 2024a).

### Instantaneous transformation of apple fruit

The CDSs of MdAAT2-like, MdMYB98-like, and MdWRKY21 were cloned into pCAMBIA 2300 and pTRV2 vectors to construct recombinant plasmids. The overexpression vectors MdAAT2-like-2300, MdMYB98-like-2300, and MdWRKY21-2300 were introduced into *A. tumefaciens* strain GV3101. Concurrently, pTRV1 was used as an auxiliary plasmid, and recombinant pTRV2 constructs (MdAAT2-like-TRV, MdMYB98-like-TRV, and MdWRKY21-TRV) were also integrated into *Agrobacterium* GV3101. The transformed bacterial cultures were resuspended in osmotic buffer and injected into the skin of ‘Golden Crown’ apples according to established methods (Wang et al. 2023a). The infected fruits were incubated in darkness for 24 h, followed by a five-day incubation under constant light (100 µmol/m^2^/s). After the incubation period, tissue surrounding the injection sites was collected for analysis of aromatic ester content and gene expression levels. The primers used for gene cloning and vector construction are listed in Table S2.

### Western blot analysis

Western blotting was performed as previously described (Wang et al. 2023a, b) using anti-Flag and anti-GFP antibodies from Abmart Medical Technology (Shanghai, China) Co., Ltd. Briefly, 0.1 g of transformed apple calli or fruit tissues were collected and ground in liquid nitrogen. The powder was homogenized in 500 μL of lysis buffer supplemented with 5.0 μL each of the protein inhibitors PMSF and cocktail. The mixture was thoroughly ground on ice and then centrifuged at 13,000 g for 10 min at 4 °C. A certain volume of the supernatant was mixed with 5 × SDS loading buffer and boiled in a water bath for 10 min. The denatured proteins were separated by SDS-PAGE and subsequently transferred to a PVDF membrane. The membrane was blocked and then incubated with primary antibodies (anti-Flag or anti-GFP) diluted 1:5000, followed by incubation with an HRP-conjugated secondary antibody diluted 1:10,000. After washing, the protein bands were visualized using a chemiluminescent substrate working solution and imaged.

### Tomato transformation

Cotyledons from the tomato variety *S. lycopersicum* cv. Micro-Tom were subjected to infection with *A. tumefaciens* strain GV3101, which carried the constructs *MdAAT2-like*−2300, *MdMYB98-like*−2300, and *MdWRKY21*−2300. After infection, explants were cultured on MS medium supplemented with 50 mg L^−1^ kanamycin to facilitate the selection of resistant shoots. Transgene-positive plants were screened, and the levels of aromatic esters were quantified in fruits harvested from T3-positive transgenic lines. Each positive transgenic line was considered a distinct biological replicate, with three replicates used for every analysis.

### Y1H screening of apple fruit cDNA library

To identify transcription factors that specifically bind to the promoters of *MdAAT2-like*, *MdMYB98-like*, and *MdWRKY21*, a Y1H screening was conducted. The 2000 bp promoter regions of these genes were cloned into the pAbAi vector to construct the bait plasmids, which were subsequently linearized and transformed into Y1H Gold yeast cells. cDNA libraries from apple fruit at various developmental stages were generated using the Clontech Matchmaker one-hybrid system and obtained from Kelong Technology Co., Ltd. (Shanghai, China). The AD-cDNA libraries were introduced into yeast cells carrying the bait constructs to screen for transcription factors with specific binding activity. Y1H screening was performed following previously described protocol (Tran et al. 2004). The primers used for vector construction and screening are listed in Supplemental Table S4.

### Y1H and Y2H assays

To investigate the regulatory interactions between *MdMYB98-like*, *MdWRKY21*, and *MdAAT2-like*, both Y1H and Y2H assays were conducted. The CDSs of *MdMYB98-like* and *MdWRKY21* were cloned into the pGADT7 vector to generate the MdMYB98-like and MdWRKY21-AD recombinant vectors, respectively. The 2000 bp promoter regions of *MdAAT2-like*, *MdMYB98-like*, and *MdWRKY21* were inserted into the pAbAi vector to construct the bait plasmids. The minimum inhibitory concentration of AbA was determined before transforming the AD prey vectors into the yeast cells containing the bait constructs. A pGADT7 empty vector served as the negative control. Yeast growth on SD/-Leu plates supplemented with AbA was assessed to determine the specific binding interactions between transcription factors and their target promoters.

To analyze potential protein–protein interactions, MdMYB98-like was cloned into the pGBKT7 vector (MdMYB98-like-pGBKT7), while MdWRKY21 was inserted into the pGADT7 vector (MdWRKY21-pGADT7). The recombinant plasmids were cotransformed into the Y2H Gold yeast strain and cultivated on SD/-Trp/-His/-Leu/-Ade medium supplemented with 200 mM AbA. Additionally, yeast cells were plated on SD/-Trp/-Leu/-His/-Ade medium containing X-α-Gal to confirm interactions based on the formation of blue colonies.

### EMSAs

To validate the direct interaction between MdMYB98-like and MdWRKY21 with their corresponding promoter sequences, EMSAs were performed. The CDSs of these proteins were cloned into the pET32a expression vector and transformed into *Escherichia coli* strains. Protein expression was induced with isopropyl β-D-1-thiogalactopyranoside (IPTG) at 16 °C while shaking at 180 rpm for 6 h. Subsequently, the expressed MdMYB98-like/MdWRKY21-His fusion proteins were purified using a Ni-agarose His-Tagged Protein Purification Kit. In the EMSA, purified proteins were incubated with biotin-labeled probes DNA designed to target the respective promoter sequences. The assay was performed using the Light Shift Chemiluminescent EMSA Kit, following the manufacturer’s instructions. Primer details for probe synthesis are provided in Table S4 (see online supplementary material).

### ChIP-qPCR analysis

ChIP-qPCR analysis was conducted following a previously reported protocol to investigate the binding of MdMYB98-like and MdWRKY21 to their target promoters (Kim & Dekker 2018). ‘Orin’ apple calli were treated with 1% formaldehyde for 10 min to crosslink DNA–protein interactions. The reaction was quenched by adding glycine, and chromatin was subsequently isolated. The extracted chromatin was sonicated to fragment genomic DNA into 0.2–0.5 kb fragments. This sonicated mixture was centrifuged at 13,000 g for 5 min at 4 °C, and the supernatant containing sheared chromatin was collected. The chromatin solution was incubated overnight at 4 °C with an anti-GFP antibody under gentle agitation. Immunoprecipitated chromatin complexes were captured using ChIP-grade protein A/G agarose beads (Thermo Fisher) for 1 h at 4 °C. After centrifugation at 1,000 g, the bound chromatin was eluted, and the crosslinking was reversed by incubating 5 M NaCl at 65 °C overnight. The recovered DNA fragments were purified and analyzed by qPCR using gene-specific primers (Supplemental Table S6). The fold-enrichment was calculated as follows: % Input = 2 ^ (Ct_Input_—Ct_ChIP_) × dilution factor (F), where F is the dilution factor of the input DNA. The final fold-enrichment over the control IgG was then calculated as (% Input_anti-GFP antibody_)/(% Input_IgG_). Each ChIP assay was performed with three technical replicates, and qPCR was conducted on three independent biological replicates to ensure reproducibility. Three technical replicates were used for each calculation. A fold-enrichment greater than 2 (P < 0.05, determined by Student's *t*-test) was considered statistically significant.

### Dual-LUC reporter assay

To investigate the regulatory effects of MdMYB98-like and MdWRKY21, their CDSs were cloned into the pGreenII 62-SK vector as effectors. Concurrently, the 2,000 bp promoter regions of *MdAAT2-like*, *MdMYB98-like*, and *MdWRKY21* were inserted into the pGreenII 0800-LUC vector, where firefly luciferase served as the reporter gene and Renilla luciferase (REN) as the internal control. The recombinant plasmids were transformed into *A. tumefaciens* strain LBA4404 and co-infiltrated with the P19 plasmid to suppress gene silencing. The transformed *A. tumefaciens* cells were resuspended in infiltration buffer and infiltrated into the leaves of *N. benthamiana*. After an incubation period of 48 h, leaf samples were collected to assess LUC activity using the Dual-Glo® Luciferase Assay Kit (Promega, Wisconsin, USA). A microplate reader (Infinite M200 PRO, Tecan, Switzerland) was used for luminescence detection. Details of the primers used for vector construction are provided in Supplemental Table S2.

### BiFC analysis

The CDS of MdMYB98-like was combined with the C-terminal section of YFP (pSPYCE), whereas MdWRKY21 was linked to the N-terminal portion of YFP (pSPYNE). The resulting recombinant vectors (MdMYB98-like-pSPYCE and MdWRKY21-pSPYNE) were introduced into *A. tumefaciens* strain GV3101 carrying the pSoup-p19 helper plasmid. A 1:1 mixture of *A. tumefaciens* suspensions containing MdMYB98-like-pSPYCE and MdWRKY21-pSPYNE was infiltrated into fresh *N. benthamiana* leaves. The plants were incubated for 24 h in the dark, followed by 36 h under normal light conditions. Fluorescence was observed using a laser confocal microscope (10 × and 20 × air objectives). The excitation wavelengths were set at 405 nm to detect the DAPI nuclear stain and 514 nm to detect the reconstituted YFP signal.

### Pull-down assay

To investigate the physical interaction between MdMYB98-like and MdWRKY21, a pull-down assay was conducted. The CDS of MdMYB98-like was cloned into the pET-32a (+) vector to generate a His-tagged fusion protein, whereas MdWRKY21 was inserted into the pGEX-4 T-1 vector for the expression of a GST fusion protein. Both recombinant vectors were introduced into the *E. coli* strain BL21 to produce MdMYB98-like-His and MdWRKY21-GST proteins. The isolation of GST fusion proteins was conducted using a protein purification kit from Beyotime Biotechnology (Shanghai, China), following the manufacturer's instructions. Eluted protein samples were analyzed via western blotting using anti-GST and anti-His antibodies (Abmart, Shanghai, China). Primer details are available in Supplemental Table S4.

### Statistical analysis

All experiments were performed in triplicate, and data are presented as mean ± standard error (SE) based on three independent biological replicates. Error bars represent standard deviations. To determine statistical significance, Student’s *t*-test was employed for comparisons between two groups, with statistical significance denoted as *P < 0.05 and **P < 0.01. For multiple-group comparisons, one-way ANOVA was utilized, followed by Tukey’s post-hoc test. Distinct letters above bars denote statistically significant differences (*P* < 0.05) among groups. All figures and graphs were created using GraphPad Prism Software (version 8.0; San Diego, CA, USA).

## Supplementary Information

Additional file 1: Fig. S1. Verification of MdAAT2-like in transiently transformed apple fruits. Fig. S2. Analysis of regulatory elements located 2000 bp upstream of the MdAAT2-like promoter. Fig. S3. Expression profile of MdMYB98-like in fruit during ripening. Fig. S4. Molecular analysis of MdMYB98-like function in transiently transformed apple fruits. Fig. S5. Sequence analysis of MdMYB98-like knockout line. Fig. S6. Characterization of MdMYB98-like in transgenic calli. Fig. S7. Verification of MdMYB98-like/MdAAT2-like Co-transformation. Fig. S8. MdWRKY21 correlation with MdAAT2-like and MdMYB98-like expression and various aromatic compound contents. Fig. S9. Identification of MdWRKY21 overexpression and knockout. Fig. S10. Changes in the relative expression of related genes and content of aromatic compounds in transgenic MdWRKY21 tomato. Fig. S11. Verification of MdWRKY21/MdAAT2-like Co-transformation. Fig. S12. Changes in aroma content after simultaneous overexpression of MdWRKY21 and MdAAT2-like. Fig. S13. The relative expression levels of MdWRKY21 in transgenic MdMYB98-like apple fruits and calli, and of MdMYB98-like in transgenic MdWRKY21 apple fruits and calli. Fig. S14. Changes in aroma content after simultaneous overexpression of MdMYB98-like and MdWRKY21. Fig. S15. Differential regulation of MdAAT genes by MdMYB98-like and MdWRKY21.

Additional file 2: Table S1. Member of AAT gene family in Malus domestica. Table S2. Activities of purified MdAAT1, MdAAT2, and MdAAT2-like proteins towards different alcohols (with Acetyl-CoA as acyl donor). Table S3. Estimated kinetic parameters (Km and Kcat/Km) of MdAAT1, MdAAT2, and MdAAT2-like proteins for key substrates. Table S4. Primers of TFs which have expression correlation with MdAAT2-like during apple fruit ripening. Table S5. Correlation analysis of candidate transcription factors with MdAAT2-like. Table S6. Other primers utilized in this study.

## Data Availability

The data underlying this article are available in the article and its supplementary material.

## Abbreviations

2,4-D: 2,4-Dichlorophenoxyacetic acid
6-BA: 6-Benzylaminopurine
AAT: Alcohol acyltransferase
AbA: Aureobasidin A
BiFC: Bimolecular fluorescence complementation
ChIP-qPCR: Chromatin immunoprecipitation
CDS: Coding sequences
cDNA: Complementary DNA
DAB: Days after flowering
DVB/CAR/PDMS: Divinylbenzene/carboxen/polydimethylsiloxane
EMSA: Electrophoretic mobility shift assay
LUC: Firefly luciferase
HS-SPME: Headspace solid-phase microextraction
IPTG: Isopropyl β-D-1-thiogalactopyranoside
LCI: Luciferase complementation imaging
MS: Murashige and Skoog
RT-qPCR: Quantitative real-time polymerase chain reaction
REN: Renilla luciferase
RNA-Seq: RNA sequencing
sgRNA: Single guide RNA
WT: Wild-type
Y1H: Yeast one-hybrid
Y2H: Yeast two-hybrid
YFP: Yellow fluorescent protein

## References

[CR1] Beekwilder J, Alvarez-Huerta M, Neef E, Verstappen F, Bouwmeester H, Aharoni A. Functional characterization of enzymes forming volatile esters from strawberry and banana. Plant Physiol. 2004;135:1865–78. 15326278 10.1104/pp.104.042580 PMC520758

[CR2] Costa F, Cappellin L, Longhi S, Gasperi F. Assessment of apple (*Malus x domestica* Borkh.) fruit texture by a combined acoustic-mechanical profiling strategy. Postharvest Biol Technol. 2011;61:21–8.

[CR3] Defilippi BG, Kader AA, Dandekar AM. Apple aroma: alcohol acyltransferase, a rate-limiting step for ester biosynthesis, is regulated by ethylene. Plant Sci. 2005;168:1199–210.

[CR4] El-Sharkawy I, Manríquez D, Flores FB, Regad F, Bouzayen M, Latché A, et al. Functional characterization of a melon alcohol acyl-transferase gene family involved in the biosynthesis of ester volatiles. Identification of the crucial role of a threonine residue for enzyme activity. Plant Mol Biol. 2005;59:345–62. 16247561 10.1007/s11103-005-8884-y

[CR5] Espino-Díaz M, Sepúlveda DR, González-Aguilar G, Olivas GI. Biochemistry of apple aroma: a review. Food Technol Biotechnol. 2016;54:375–97. 28115895 10.17113/ftb.54.04.16.4248 PMC5253989

[CR6] Goulet C, Kamiyoshihara Y, Lam NB, Richard T, Taylor MG, Tieman DM, et al. Divergence in the enzymatic activities of a tomato and *Solanum pennellii* alcohol acyltransferase impacts fruit volatile ester composition. Mol Plant. 2015;8:153–62. 25578279 10.1016/j.molp.2014.11.007

[CR7] Hellens RP, Allan AC, Friel EN. Transient expression vectors for functional genomics, quantification of promoter activity and RNA silencing in plants. Plant Methods. 2005;1:13. 16359558 10.1186/1746-4811-1-13 PMC1334188

[CR8] Hong GJ, Xue XY, Mao YB, Wang LJ, Chen XY. Arabidopsis MYC2 interacts with DELLA proteins in regulating sesquiterpene synthase gene expression. Plant Cell. 2012;24:2635–48. 22669881 10.1105/tpc.112.098749 PMC3406894

[CR9] Jian W, Cao H, Yuan S, Liu Y, Lu J, Lu W, et al. SlMYB75, an MYB-type transcription factor,promotes anthocyanin accumulation and enhances volatile aroma production in tomato fruits. Hortic Res. 2019;6:22. 30729012 10.1038/s41438-018-0098-y PMC6355774

[CR10] Kim TH, Dekker J. ChIP-Quantitative Polymerase Chain Reaction (ChIP-qPCR). Cold Spring Harbor Protoc. 2018;2018(5). 10.1101/pdb.prot082628. 10.1101/pdb.prot082628 29717044

[CR11] Li D, Xu Y, Xu G, Gu L, Li D, Shu H. Molecular cloning and expression of a gene encoding alcohol acyltransferase (*MdAAT2*) from apple (cv. Golden Delicious). Phytochemistry. 2006a;67:658–67. 16524607 10.1016/j.phytochem.2006.01.027

[CR12] Li DP, Xu YF, Sun LP, Liu LX, Shu HR. Salicylic acid, ethephon, and methyl jasmonate enhance ester regeneration in 1-MCP-treated apple fruit after long-term cold storage. J Agric Food Chem. 2006b;54:3887–95. 16719511 10.1021/jf060240j

[CR13] Li PC, Yu SW, Shen J, Li QQ, Li DP, Li DQ, et al. The transcriptional response of apple alcohol acyltransferase (*MdAAT2*) to salicylic acid and ethylene is mediated through two apple MYB TFs in transgenic tobacco. Plant Mol Biol. 2014;85:627–38. 24893956 10.1007/s11103-014-0207-8

[CR14] Li LX, Fang Y, Li D, Zhu ZH, Zhang Y, Tang ZY, et al. Transcription factors MdMYC2 and MdMYB85 interact with ester aroma synthesis gene *MdAAT1* in apple. Plant Physiol. 2023;193:2442–58. 37590971 10.1093/plphys/kiad459

[CR15] Lin XH, Li SJ, Shi YN, Ma YC, Li YC, Tan HB, et al. CitGATA7 interact with histone acetyltransferase CitHAG28 to promote citric acid degradation by regulating the glutamine synthetase pathway in citrus. Mol Hortic. 2025;5:8. 39891226 10.1186/s43897-024-00126-y PMC11786515

[CR16] Liu H, Cao X, Liu X, Xin R, Wang JJ, Gao J, et al. UV-B irradiation differentially regulates terpene synthases and terpene content of peach. Plant Cell Environ. 2017;40:2261–75. 28722114 10.1111/pce.13029

[CR17] Liu X, Hao N, Feng R, Meng Z, Li Y, Zhao Z. Transcriptome and metabolite profiling analyses provide insight into volatile compounds of the apple cultivar “Ruixue” and its parents during fruit development. BMC Plant Biol. 2021;21:231. 34030661 10.1186/s12870-021-03032-3 PMC8147058

[CR18] Liu H, Gao J, Sun J, Li S, Zhang BF, Wang ZW, et al. Dimerization of *PtrMYB074* and *PtrWRKY19* mediates transcriptional activation of *PtrbHLH186* for secondary xylem development in *Populus trichocarpa*. New Phytol. 2022;234:918–33. 35152419 10.1111/nph.18028 PMC9314101

[CR19] Liu X, Liu L, Yu J, Li S, Li D, Feng Y, Zhao Z. of the transcription factor *MdABF2* in abscisic acid-induced volatile compound biosynthesis in 'Ruixue' (*Malus* × *domestica*) apple during storage. 2023;205:112524.

[CR20] Livak KJ, Schmittgen TD. Analysis of relative gene expression data using real-time quantitative PCR and the 2(-delta delta C(T)) method. Methods. 2001;25:402–8. 11846609 10.1006/meth.2001.1262

[CR21] Nieuwenhuizen NJ, Chen X, Wang MY, Matich AJ, Perez RL, Allan AC, et al. Natural variation in monoterpene synthesis in kiwifruit: transcriptional regulation of terpene synthases by NAC and ETHYLENE-INSENSITIVE3-like transcription factors. Plant Physiol. 2015;167:1243. 25649633 10.1104/pp.114.254367 PMC4378164

[CR22] Niu Y, Wang R, Xiao Z, Zhu J, Sun X, Wang P. Characterization of ester odorants of apple juice by gas chromatography-olfactometry, quantitative measurements, odour threshold, aroma intensity and electronic nose. Food Res Int. 2019;120:92–101. 31000313 10.1016/j.foodres.2019.01.064

[CR23] Qin G, Wang Y, Cao B, Wang W, Tian S. Unraveling the regulatory network of the MADS box transcription factor RIN in fruit ripening. Plant J. 2012;70:243–55. 22098335 10.1111/j.1365-313X.2011.04861.x

[CR24] Souleyre EJ, Greenwood DR, Friel EN, Karunairetnam S, Newcomb RD. An alcohol acyl transferase from apple (cv. Royal Gala), MpAAT1, produces esters involved in apple fruit flavor. FEBS J. 2005;272(12):3132–44. 10.1111/j.1742-4658.2005.04732.x. 10.1111/j.1742-4658.2005.04732.x 15955071

[CR25] Souleyre EJF, Chagné D, Chen X, Tomes S, Turner RM, Wang MY, et al. The AAT1 locus is critical for the biosynthesis of esters contributing to “ripe apple” flavour in “Royal Gala” and “Granny Smith” apples. Plant J. 2014;78:903–15. 24661745 10.1111/tpj.12518

[CR26] Tran LS, Nakashima K, Sakuma Y, Simpson SD, Fujita Y, Maruyama K, et al. Isolation and functional analysis of Arabidopsis stress-inducible NAC transcription factors that bind to a drought-responsive cis-element in the early responsive to dehydration stress 1 promoter. Plant Cell. 2004;16:2481–98. 15319476 10.1105/tpc.104.022699 PMC520947

[CR27] Wang J, Luca VD. The biosynthesis and regulation of biosynthesis of Concord grape fruit esters, including “foxy” methylanthranilate. Plant J. 2010;44:606–19. 10.1111/j.1365-313X.2005.02552.x 16262710

[CR28] Wang H, Zhang SH, Fu QQ, Wang ZD, Liu XJ, Sun LL, et al. Transcriptomic and metabolomic analysis reveals a protein module involved in preharvest apple peel browning. Plant Physiol. 2023a;192:2102–22. 36722358 10.1093/plphys/kiad064

[CR29] Wang JH, Gu KD, Zhang QY, Yu JQ, Wang CK, You CX, et al. Ethylene inhibits malate accumulation in apple by transcriptional repression of aluminum-activated malate transporter 9 via the WRKY31-ERF72 network. New Phytol. 2023b;239:1014–34. 36747049 10.1111/nph.18795

[CR30] Wang JH, Sun Q, Ma CN, Wei MM, Wang CK, Zhao YW, et al. MdWRKY31-MdNAC7 regulatory network: orchestrating fruit softening by modulating cell wall-modifying enzyme MdXTH2 in response to ethylene signalling. Plant Biotechnol J. 2024;22:3244–61. 39180170 10.1111/pbi.14445 PMC11606422

[CR31] Wang S, Shi M, Zhang Y, Pan Z, Xie X, Zhang L, Sun P, Feng H, Xue H, Fang C, Zhao J. The R2R3-MYB transcription factor FaMYB63 participates in regulation of eugenol production in strawberry. Plant Physiol. 2022;188(4):2146–65. 10.1093/plphys/kiac014. 10.1093/plphys/kiac014 PMC8968321 35043961

[CR32] Zhang J, Wang Y, Zhang SS, Liu WJ, Wang N, Fang HC, et al. ABIOTIC stress gene 1 mediates aroma volatiles accumulation by activating *MdLOX1a* in apple. Hortic Res. 2024a;11:uhae215. 39391012 10.1093/hr/uhae215 PMC11464680

[CR33] Zhang RX, Liu Y, Zhang X, Chen XM, Sun JJ, Zhao Y, et al. Two adjacent NAC transcription factors regulate fruit maturity date and flavor in peach. New Phytol. 2024b;241:632–49. 37933224 10.1111/nph.19372

[CR34] Zhang Z, Chen C, Jiang C, Lin H, Zhao Y, Guo Y. VvWRKY5 positively regulates wounding-induced anthocyanin accumulation in grape by interplaying with *VvMYBA1* and promoting jasmonic acid biosynthesis. Hortic Res. 2024c;11:uhae083. 38766531 10.1093/hr/uhae083 PMC11101322

[CR35] Zhu F, Wen WW, Cheng YJ, Fernie AR. The metabolic changes that effect fruit quality during tomato fruit ripening. Mol Hortic. 2022;2:2. 37789428 10.1186/s43897-022-00024-1 PMC10515270

