# Supplementary material for: A MYB–WRKY feedback module activates MdAAT2‑like to regulate aromatic ester biosynthesis during apple ripening
Source: Mol Hortic. 2026 Aug 3;6:56. doi: 10.1186/s43897-026-00240-z (PMC13430851; doi:10.1186/s43897-026-00240-z)
Supplement: Supplementary file 1 — Additional file 1: Fig. S1. Verification of MdAAT2-like in transiently transformed apple fruits. Fig. S2. Analysis of regulatory elements located 2000 bp upstream of the MdAAT2-like promoter. Fig. S3. Expression profile of MdMYB98-like in fruit during ripening. Fig. S4. Molecular analysis of MdMYB98-like function in transiently transformed apple fruits. Fig. S5. Sequence analysis of MdMYB98-like knockout line. Fig. S6. Characterization of MdMYB98-like in transgenic calli. Fig. S7. Verification of MdMYB98-like/MdAAT2-like Co-transformation. Fig. S8. MdWRKY21 correlation with MdAAT2-like and MdMYB98-like expression and various aromatic compound contents. Fig. S9. Identification of MdWRKY21 overexpression and knockout. Fig. S10. Changes in the relative expression of related genes and content of aromatic compounds in transgenic MdWRKY21 tomato. Fig. S11. Verification of MdWRKY21/MdAAT2-like Co-transformation. Fig. S12. Changes in aroma content after simultaneous overexpression of MdWRKY21 and MdAAT2-like. Fig. S13. The relative expression levels of MdWRKY21 in transgenic MdMYB98-like apple fruits and calli, and of MdMYB98-like in transgenic MdWRKY21 apple fruits and calli. Fig. S14. Changes in aroma content after simultaneous overexpression of MdMYB98-like and MdWRKY21. Fig. S15. Differential regulation of MdAAT genes by MdMYB98-like and MdWRKY21. [file 43897_2026_240_MOESM1_ESM.docx]

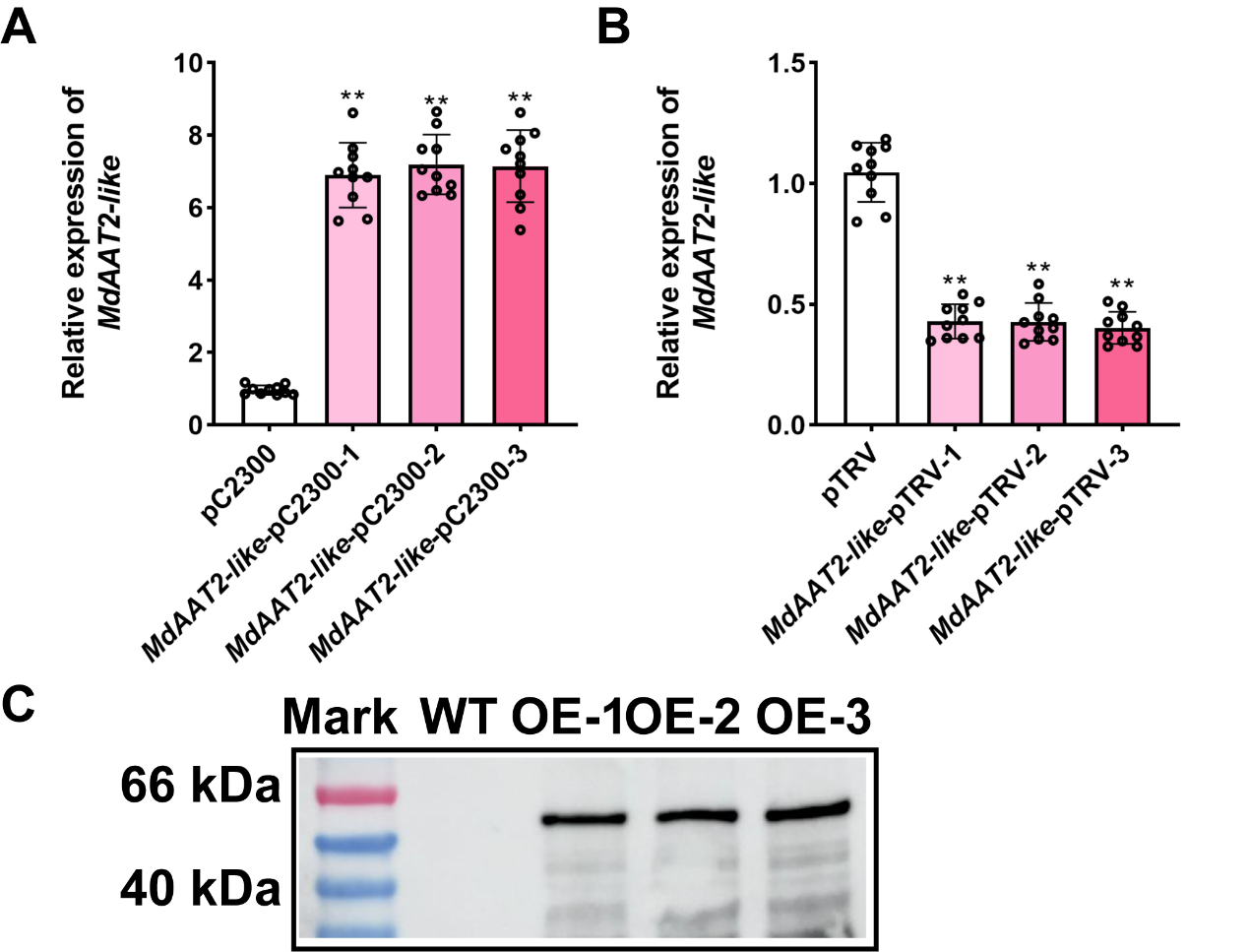


**Fig. S1.** Verification of MdAAT2-like in transiently transformed apple fruits.
**A** Relative expression of *MdAAT2-like* in apple fruits with transient overexpression. **B** Relative expression of *MdAAT2-like* in apple fruits with transient silencing. **C** Protein accumulation of MdAAT2-like in apple fruits with transient overexpression. Data are expressed as mean ± standard error (SE; n ≥ 3); ** indicates P < 0.01 (Student's *t*-test).


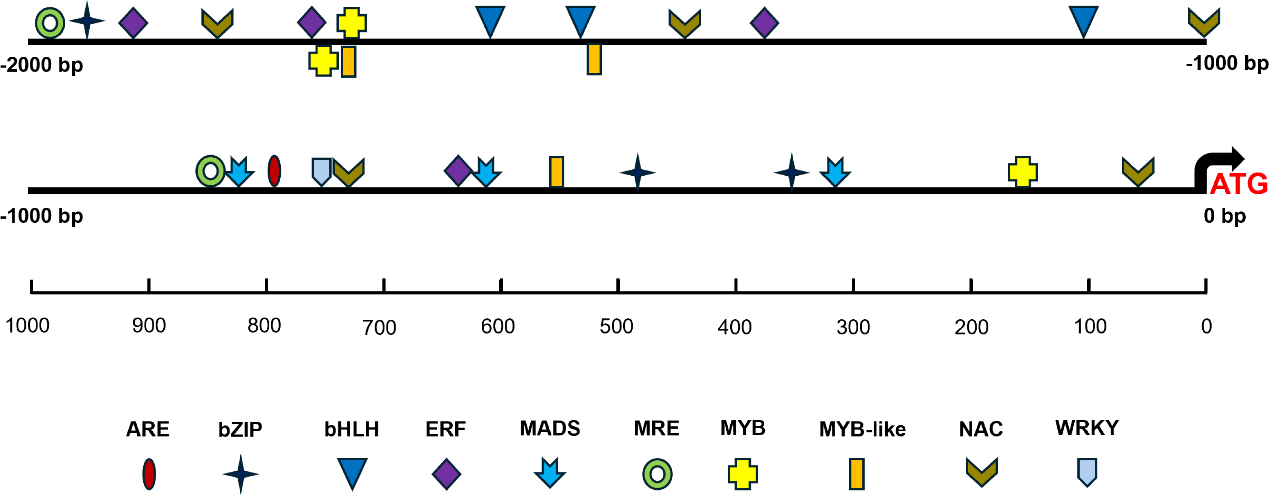


**Fig. S2.** Analysis of regulatory elements located 2000 bp upstream of the *MdAAT2-like* promoter.

Different symbols represent distinct elements within the promoter region.


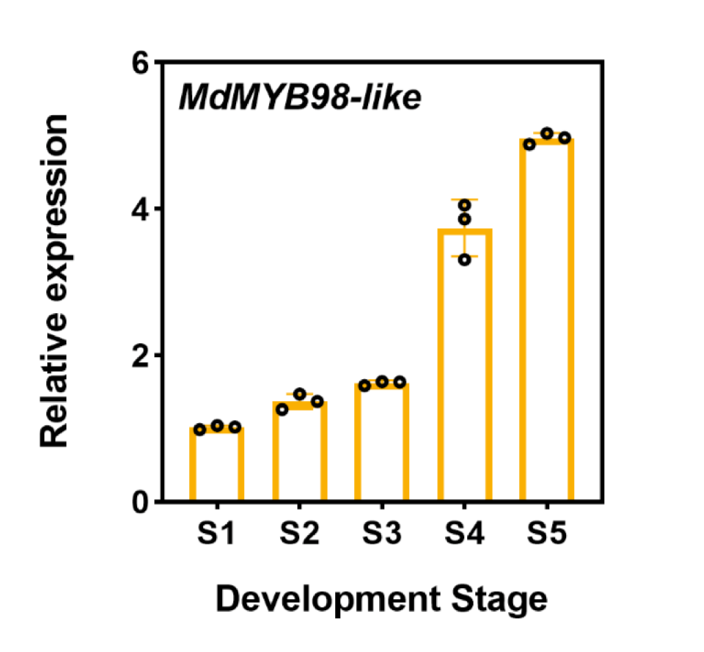


**Fig. S3.** Expression profile of *MdMYB98-like* in fruit during ripening. Data are expressed as mean ± standard error (SE; n = 3)


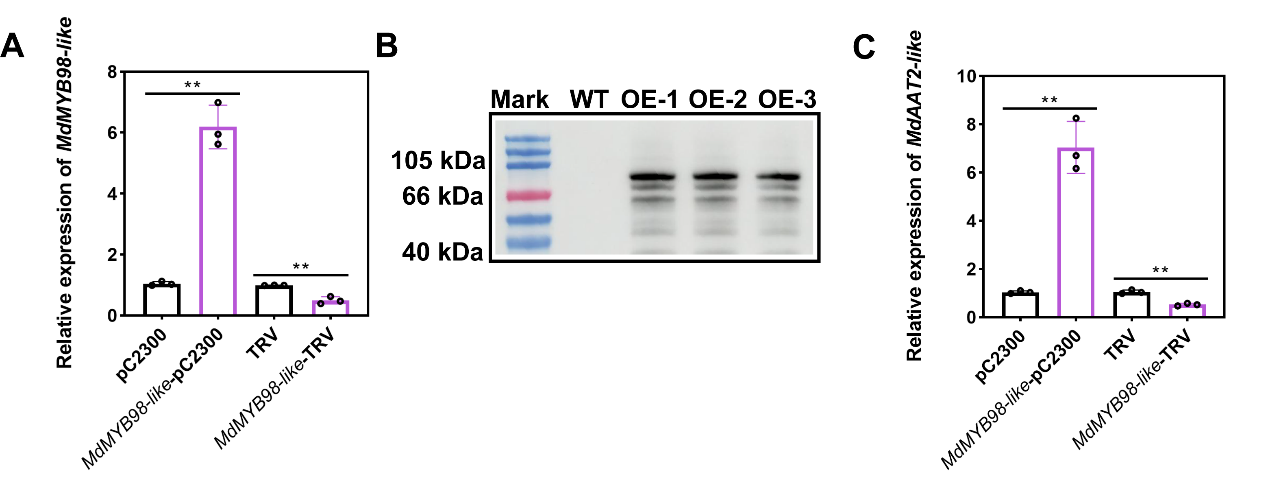


**Fig. S4.** Molecular analysis of *MdMYB98-like* function in transiently transformed apple fruits.

**A** Relative expression of *MdMYB98-like* in apple fruits following its transient expression. **B** Protein accumulation of MdMYB98-like detected by Western blot. **C** Relative expression of *MdAAT2-like* following transient expression of *MdMYB98-like*. Error bars indicate mean ± standard error (SE; n = 3), ** indicates significant differences at P < 0.01 (Student's *t*-test).


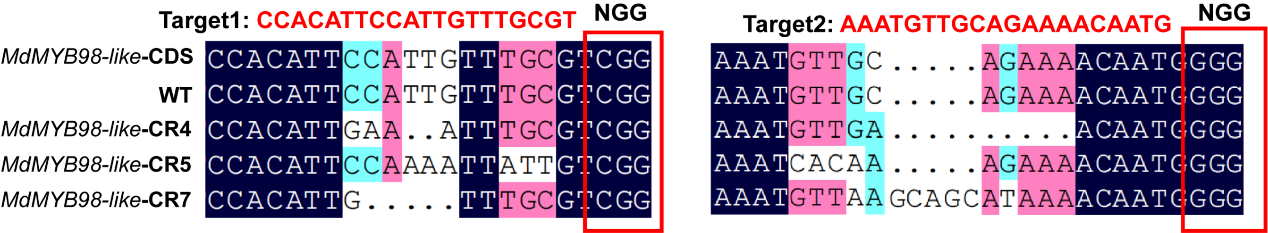


**Fig. S5.** Sequence analysis of *MdMYB98-like* knockout line.


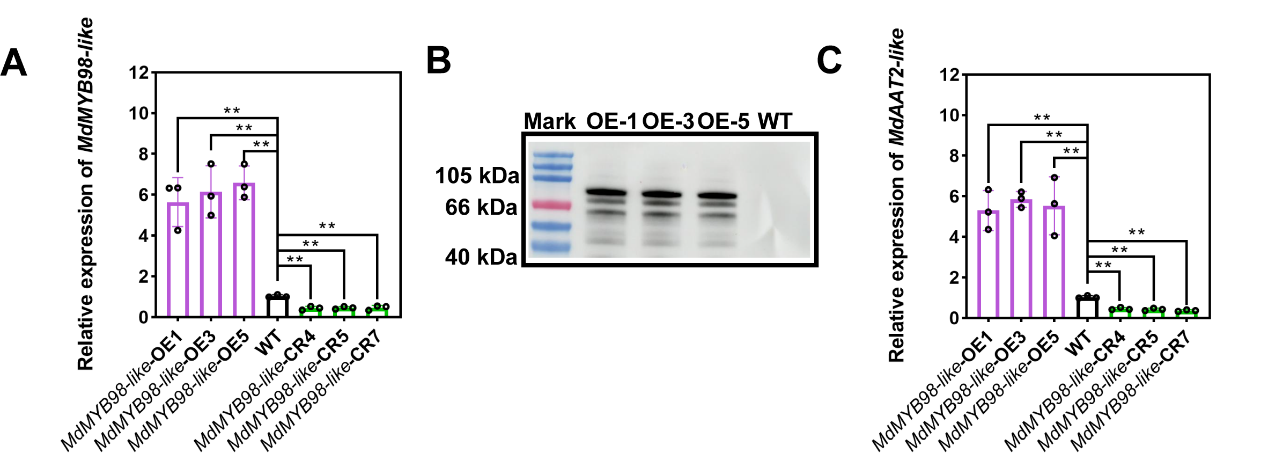


**Fig. S6.** Characterization of *MdMYB98-like* in transgenic calli.

**A** Expression levels of *MdMYB98-like* after its overexpression and knockout. **B** Protein accumulation of *MdMYB98-like* in overexpression calli by Western blot. **C** Relative expression of *MdAAT2-like* after stable expression of *MdMYB98-like*. Data are expressed as mean ± standard error (SE; n = 3); ** indicates P < 0.01 (Student's *t*-test).


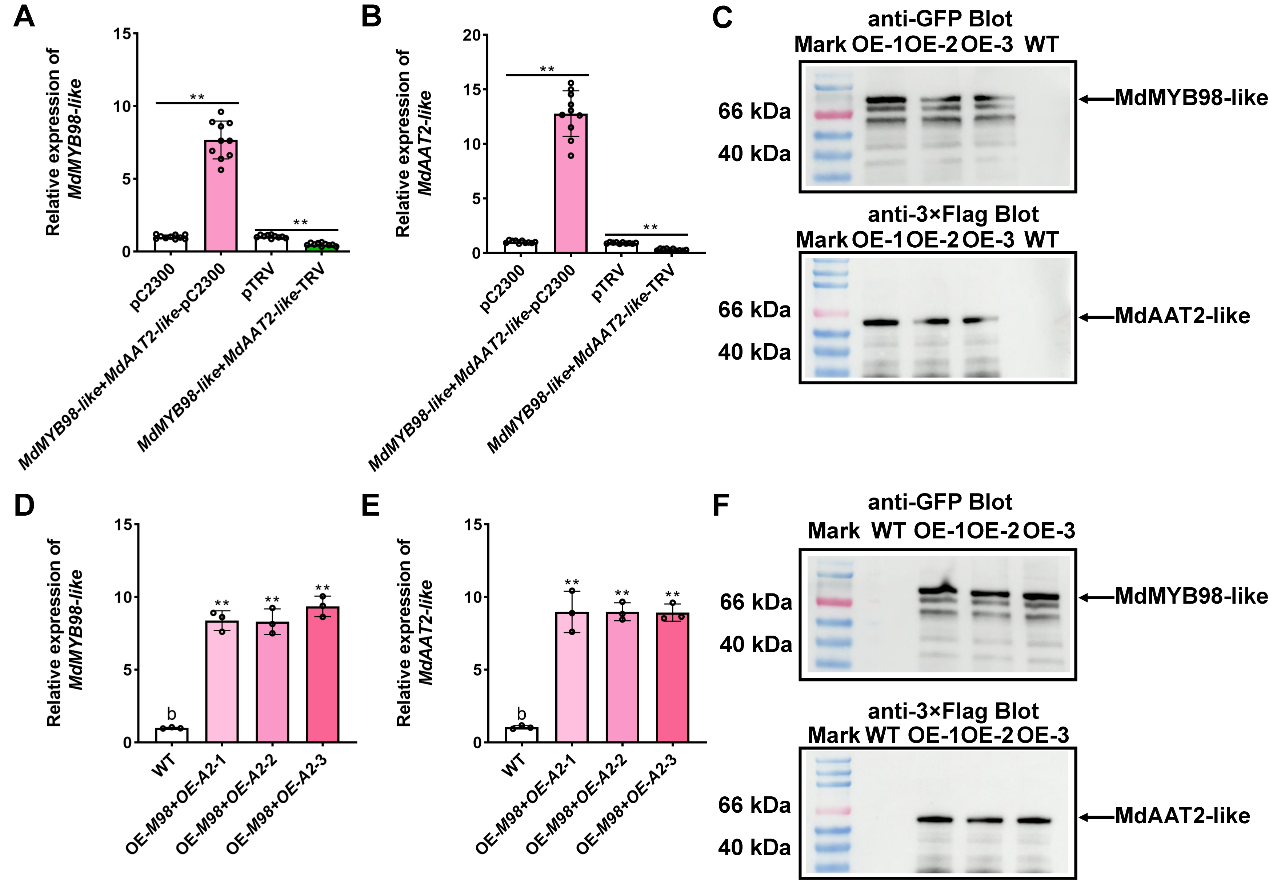


**Fig. S7.** Verification of MdMYB98-like/MdAAT2-like Co-transformation.

Relative expression of *MdMYB98-like* (**A**) and *MdAAT2-like* (**B**) in apple fruits following transient co-expression. **C** Protein accumulation of MdMYB98-like and MdAAT2-like in apple fruits with transient co-expression, analyzed by Western blot. Relative expression of *MdMYB98-like* (**D**) and *MdAAT2-like* (**E**) in calli with stable co-expression. **F** Protein accumulation of MdMYB98-like and MdAAT2-like in calli with stable co-expression, analyzed by Western blot. Data are expressed as mean ± standard error (SE; n = 3); ** indicates P < 0.01 (Student's *t*-test).


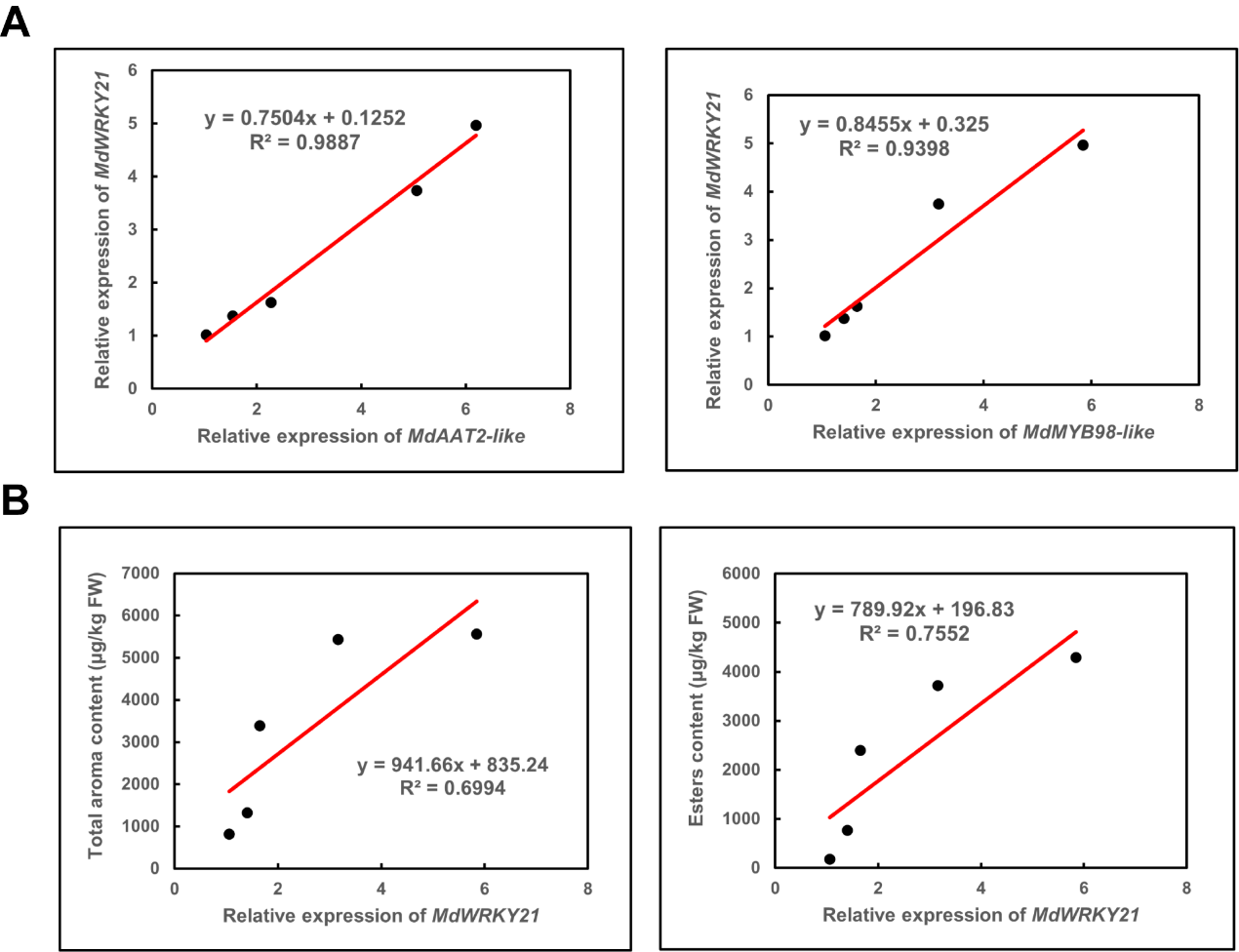


**Fig. S8.** *MdWRKY21* correlation with *MdAAT2-like* and *MdMYB98-like* expression (**A**) and various aromatic compound contents (**B**).


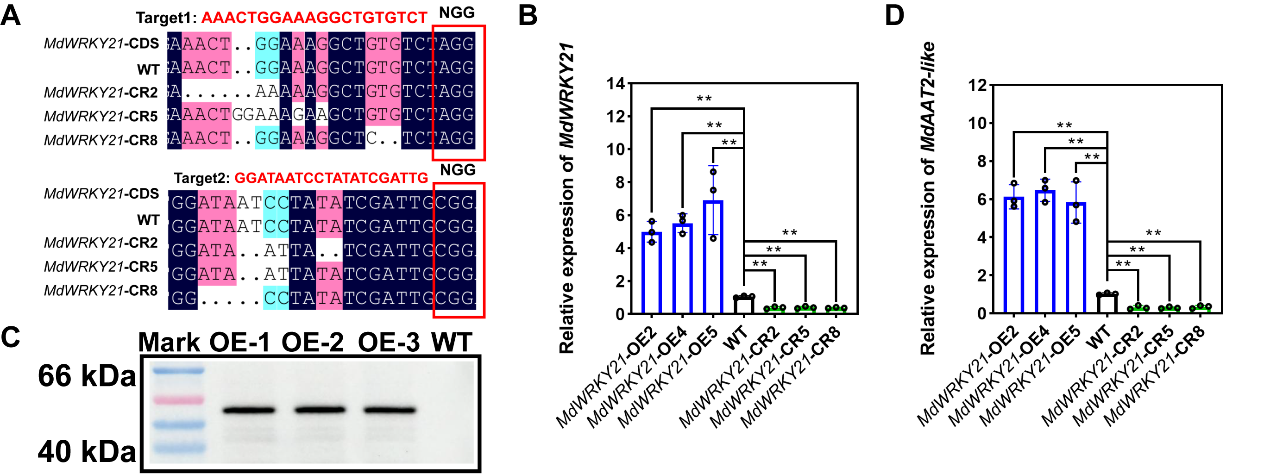


**Fig. S9.** Identification of *MdWRKY21* overexpression and knockout. **A** Sequence analysis of *MdWRKY21* stable knockout. *MdWRKY21* RNA (**B**) and protein (**C**) levels in stable overexpressing lines. **D** Relative expression of *MdAAT2-like* after *MdWRKY21* overexpression and knockout. Data are expressed as mean ± standard error (SE; n = 3); ** indicates P < 0.01, (Student's *t*-test).


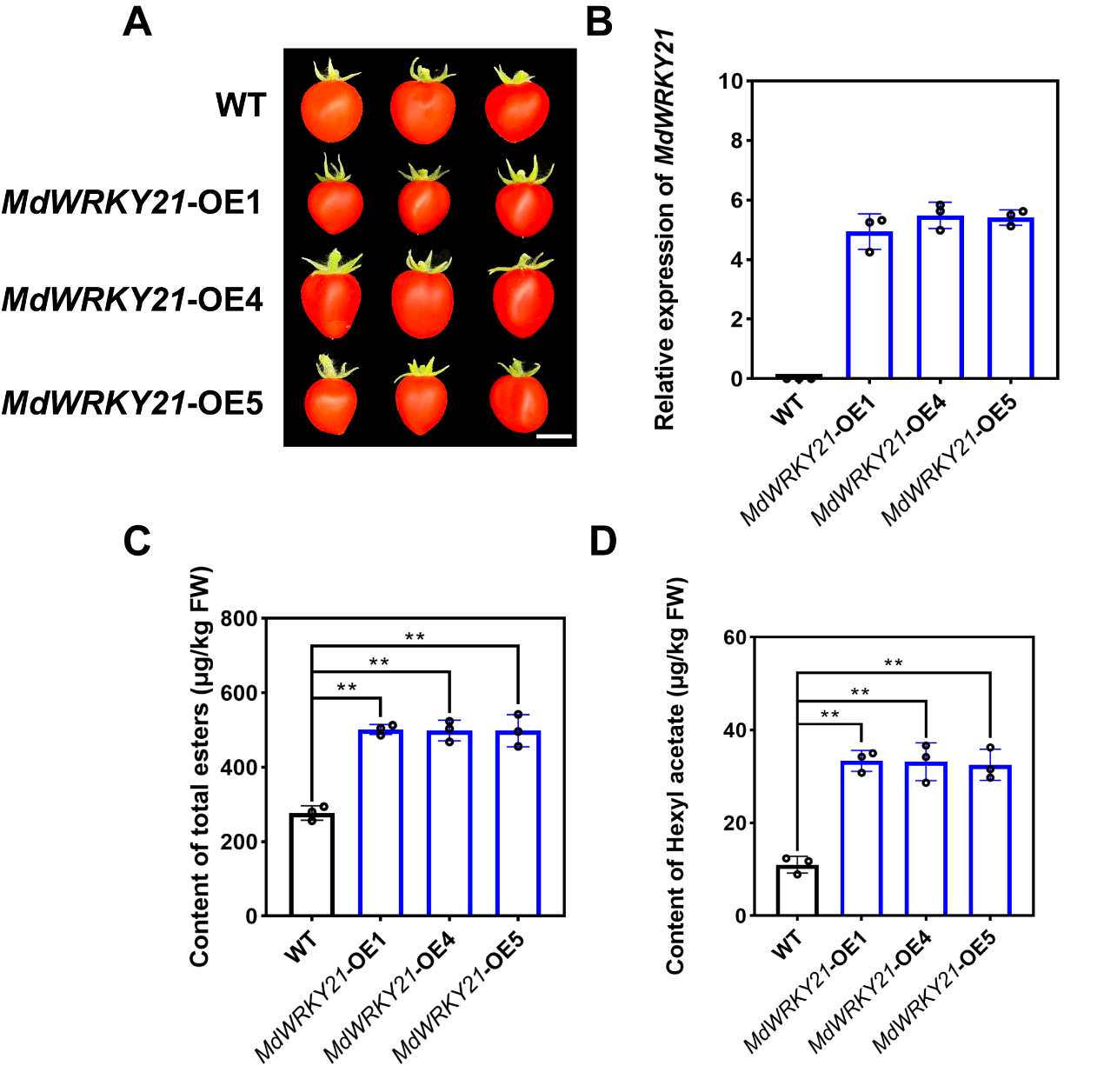


**Fig. S10.** Changes in the relative expression of related genes and content of aromatic compounds in transgenic *MdWRKY21* tomato. Bars = 1 cm. **A** Phenotype of *MdWRKY21* transgenic tomato. **B** Relative expression of *MdWRKY21* in tomatoes overexpressing *MdWRKY21*. Total ester aromatics (**C**) and hexyl acetate content (**D**) in transgenic tomato fruits. Data are expressed as mean ± standard error (SE; n = 3); ** indicates P < 0.01, (Student's *t*-test). FW, fresh weight.


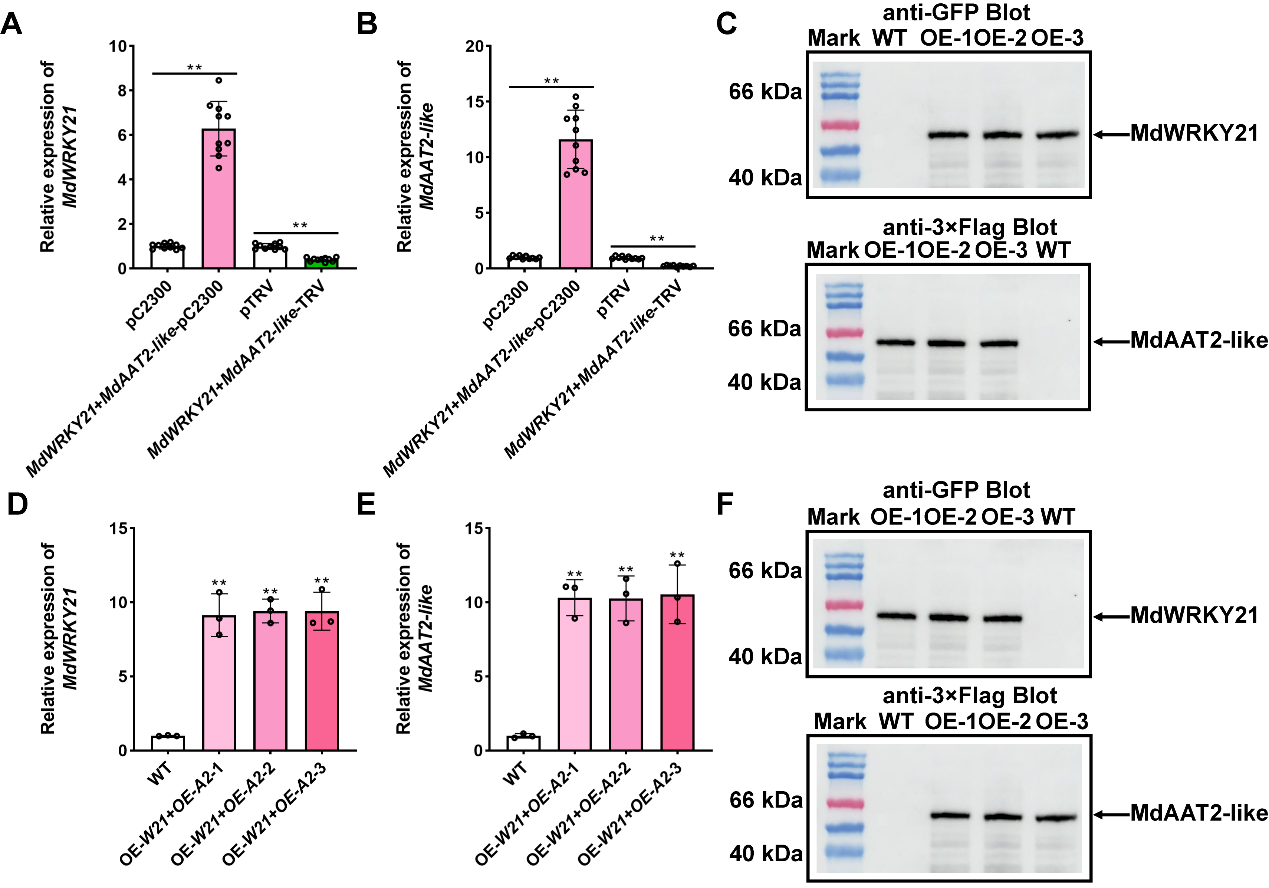


**Fig. S11.** Verification of MdWRKY21/MdAAT2-like Co-transformation.

Relative expression of *MdWRKY21* (**A**) and *MdAAT2-like* (**B**) in apple fruits following transient co-expression. **C** Protein accumulation of MdWRKY21 and MdAAT2-like in apple fruits with transient co-expression, analyzed by Western blot. Relative expression of *MdWRKY21* (**D**) and *MdAAT2-like* (**E**) in calli with stable co-expression. **F** Protein accumulation of MdWRKY21 and MdAAT2-like in calli with stable co-expression, analyzed by Western blot. Data are expressed as mean ± standard error (SE; n = 3); ** indicates P < 0.01 (Student's *t*-test).


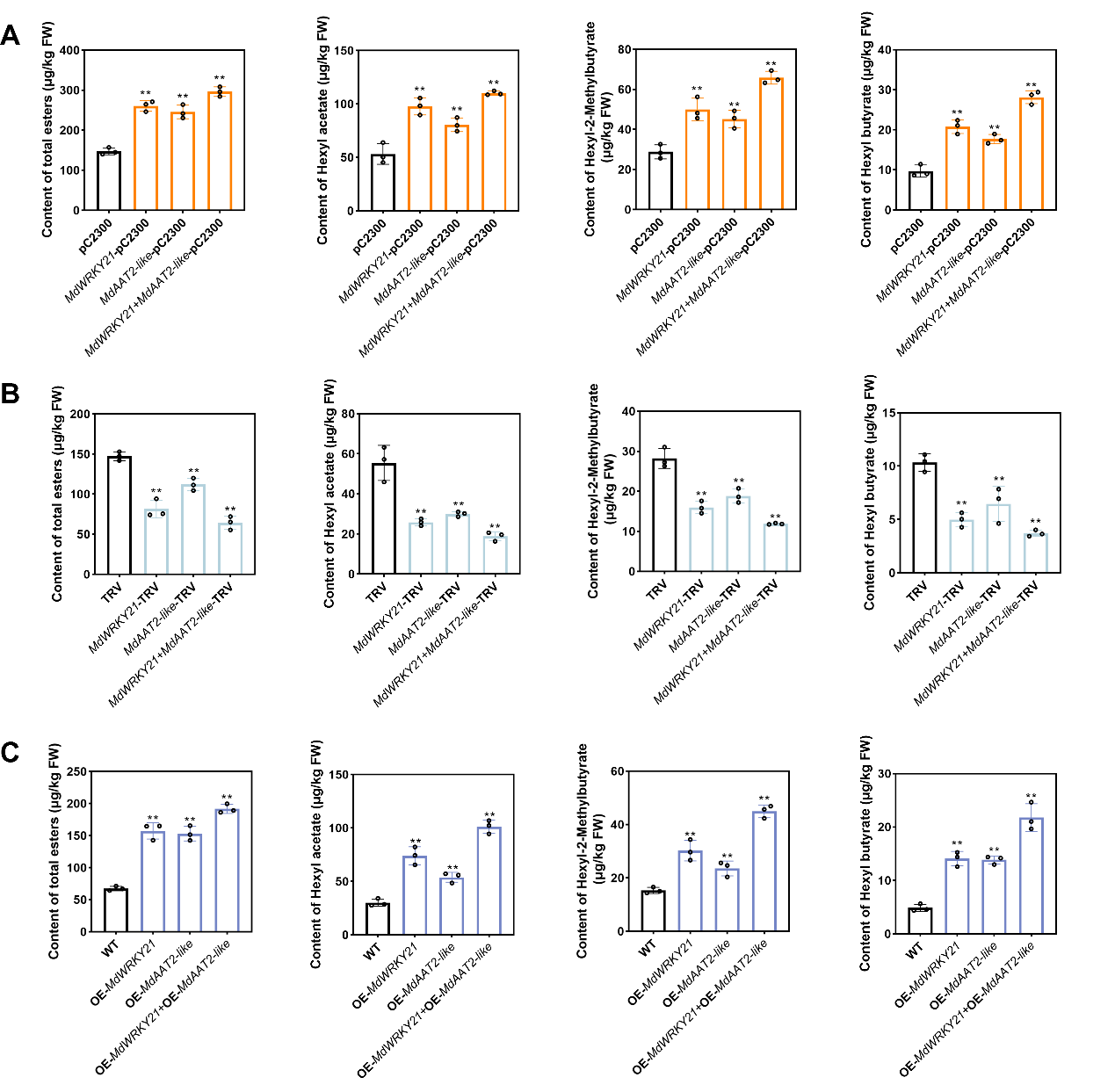


**Fig. S12.** Changes in aroma content after simultaneous overexpression of *MdWRKY21* and *MdAAT2-like*. **A** Ester content in apple fruit overexpressing *MdWRKY21*, *MdAAT2-like* and co-expressing *MdWRKY21* + *MdAAT2-like*. **B** Ester content of apple fruits silencing *MdWRKY21*, *MdAAT2-like* and co-silencing *MdWRKY21*+*MdAAT2-like*. **C** Aroma content of apple calli overexpressing *MdWRKY21*, *MdAAT2-like* and co-expressing *MdWRKY21* +*MdAAT2-like*. Data are expressed as mean ± standard error (SE; n = 3); ** indicates P < 0.01, (Student's *t*-test). FW, fresh weight.


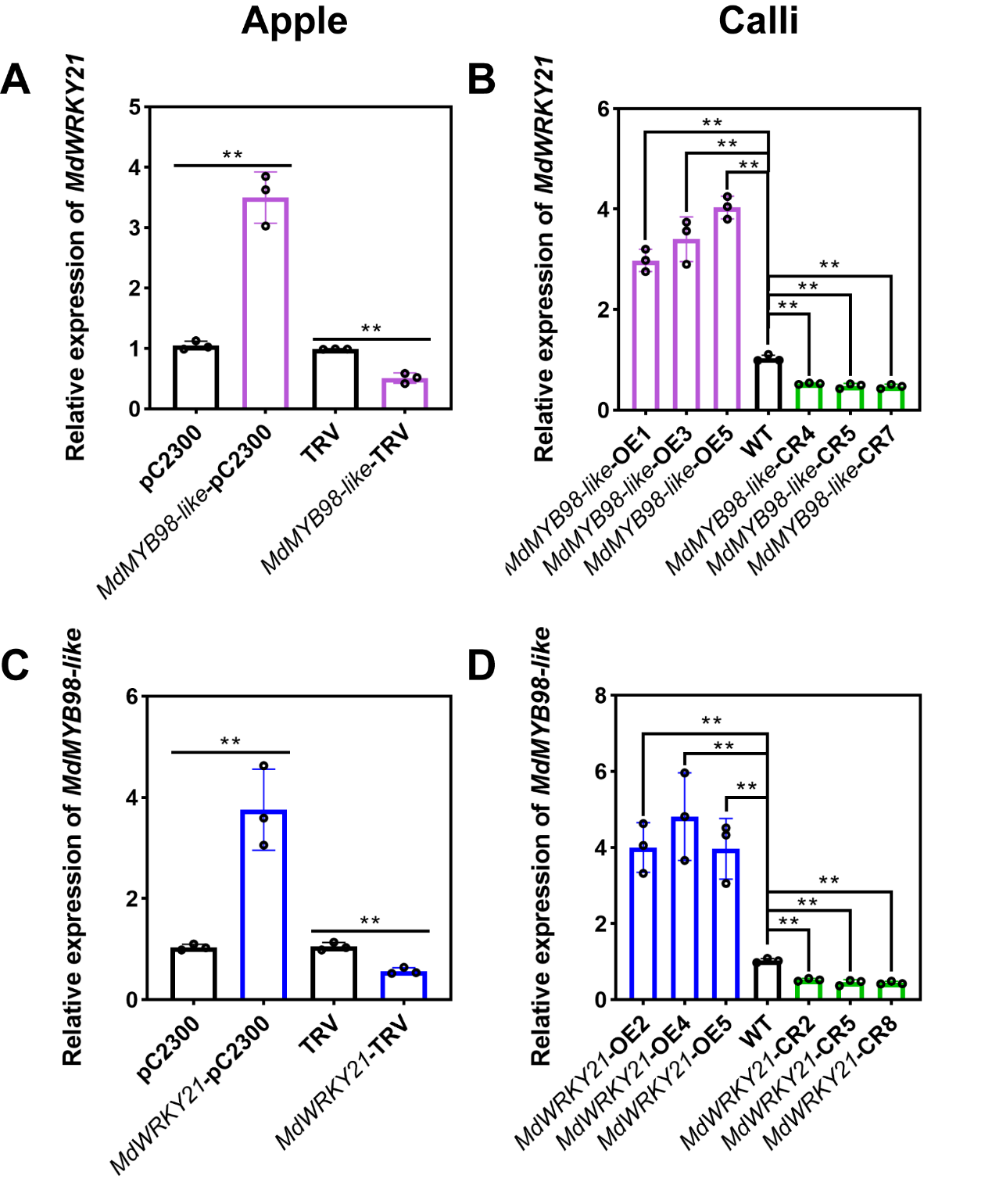


**Fig. S13.** The relative expression levels of *MdWRKY21* in transgenic *MdMYB98-like* apple fruits (**A**) and calli (**B**) and of *MdMYB98-like* in transgenic *MdWRKY21* apple fruits (**C**) and calli (**D**). Data are expressed as mean ± standard error (SE; n = 3); ** indicates P < 0.01, (Student's *t*-test).


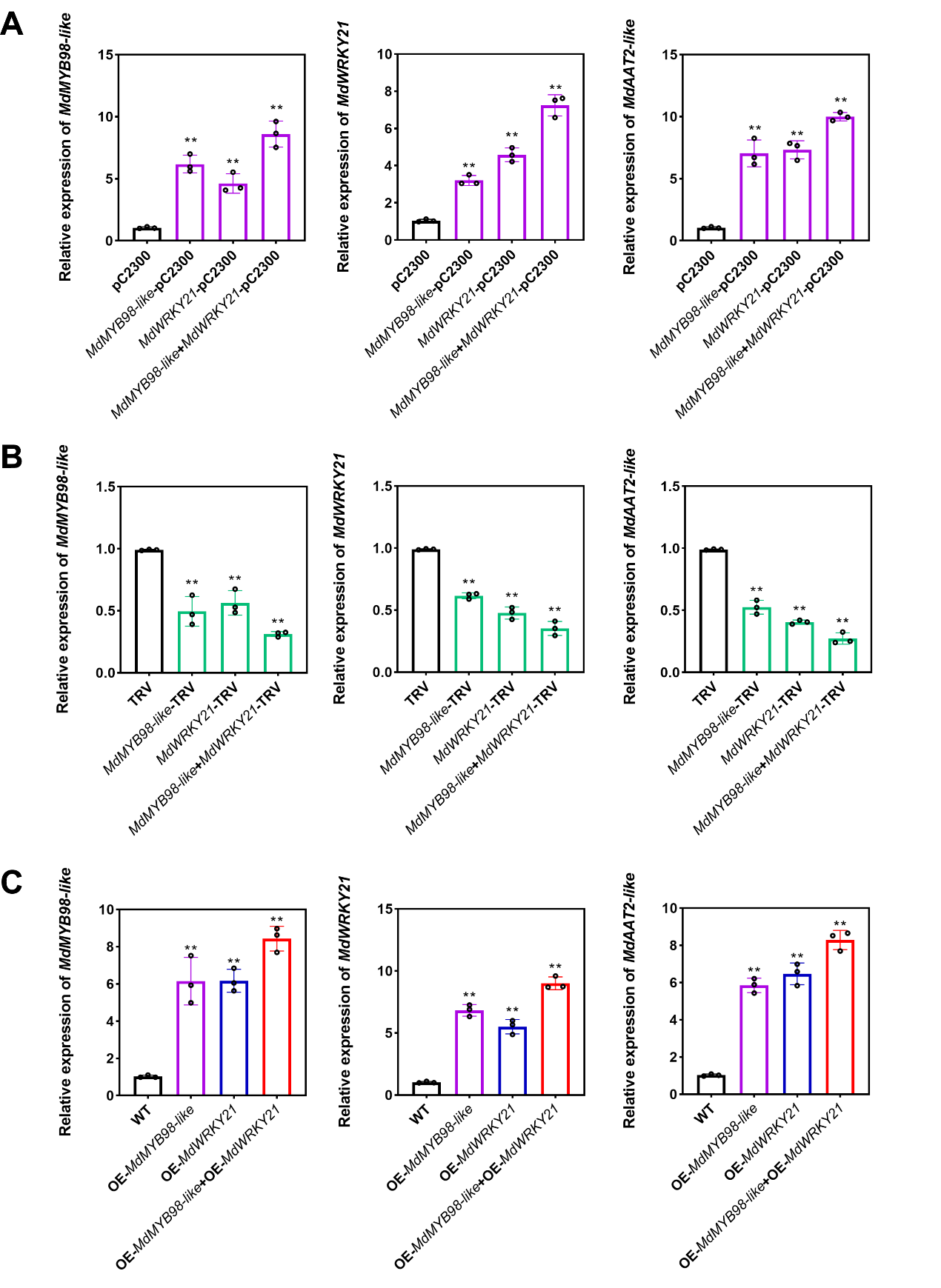


**Fig. S14.** Changes in aroma content after simultaneous overexpression of *MdMYB98-like* and *MdWRKY21*. **A** Related gene expression in apple fruit overexpressing *MdMYB98-like*, *MdWRKY21* and co-expressing *MdMYB98-like* + *MdWRKY21*. **B** Expression of related genes in fruits silencing *MdMYB98-like*, *MdWRKY21* and co-expressing *MdMYB98-like* + *MdWRKY21*. **C** Related gene expression overexpressing *MdMYB98-like*, *MdWRKY21* and co-expressing *MdMYB98-like* + *MdWRKY21*. Data are expressed as mean ± standard error (SE; n = 3); ** indicates P < 0.01, (Student's *t*-test).


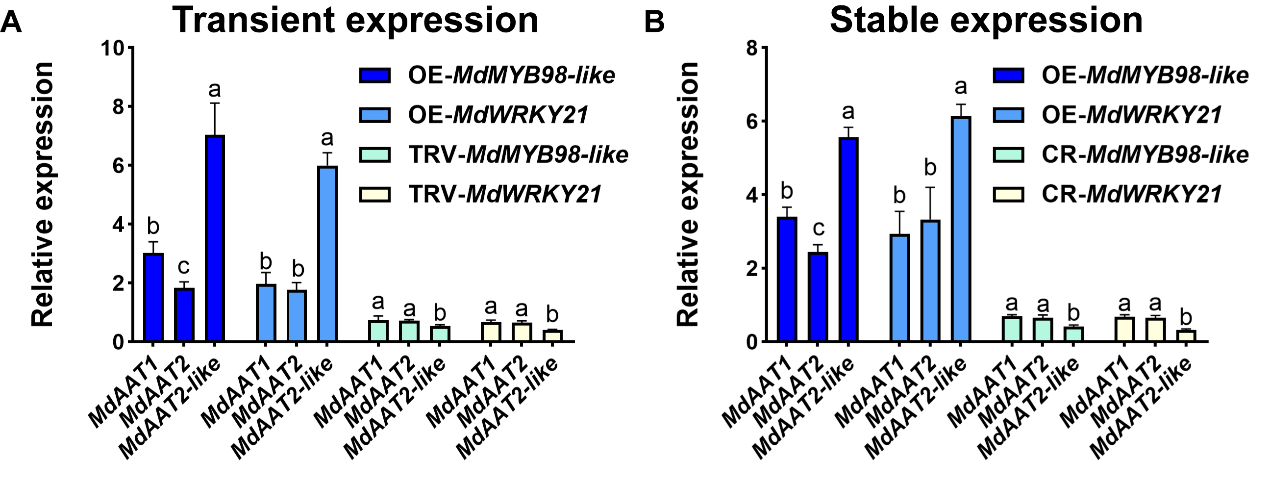


**Fig. S15.** Differential regulation of *MdAAT* genes by *MdMYB98-like* and *MdWRKY21*.

Relative expression of *MdAAT1*, *MdAAT2*, and *MdAAT2-like* in response to (**A**) transient and (**B**) stable expression of *MdMYB98-like* or *MdWRKY21*. Values with different superscript letters (a, b, c) across groups indicate significant differences at P < 0.05 as determined by Tukey's HSD test.
