# Supplementary material for: A MYB–WRKY feedback module activates MdAAT2‑like to regulate aromatic ester biosynthesis during apple ripening
Source: Mol Hortic. 2026 Aug 3;6:56. doi: 10.1186/s43897-026-00240-z (PMC13430851; doi:10.1186/s43897-026-00240-z)
Supplement: Supplementary file 2 — Additional file 2: Table S1. Member of AAT gene family in Malus domestica. Table S2. Activities of purified MdAAT1, MdAAT2, and MdAAT2-like proteins towards different alcohols (with Acetyl-CoA as acyl donor). Table S3. Estimated kinetic parameters (Km and Kcat/Km) of MdAAT1, MdAAT2, and MdAAT2-like proteins for key substrates. Table S4. Primers of TFs which have expression correlation with MdAAT2-like during apple fruit ripening. Table S5. Correlation analysis of candidate transcription factors with MdAAT2-like. Table S6. Other primers utilized in this study. [file 43897_2026_240_MOESM2_ESM.docx]

**Table S1.** Member of AAT gene family in *Malus domestica*.

| Gene ID | CDS (bp) | Protein length | MW (KDa) | PI | Subcellular Localization |
| --- | --- | --- | --- | --- | --- |
| gene:MD06G1016200 | 1488 | 495 | 55877.03 | 7.86 | Cytoplasm |
| gene:MD02G1014800 | 1380 | 459 | 51183.55 | 8.08 | Cytoplasm |
| gene:MD02G1015200 | 1368 | 455 | 50919.85 | 8.1 | Cytoplasm |
| gene:MD02G1013900 | 1380 | 459 | 51291.71 | 8.63 | Cytoplasm |
| gene:MD06G1020700 | 1380 | 459 | 51392.86 | 8.45 | Cytoplasm |
| gene:MD06G1018700 | 1428 | 475 | 53488.22 | 7.85 | Cytoplasm |
| gene:MD06G1018100 | 1383 | 460 | 51700.29 | 8.31 | Cytoplasm |
| gene:MD06G1020400 | 1383 | 460 | 51702.21 | 8.31 | Cytoplasm |
| gene:MD03G1080200 | 1365 | 454 | 50539.48 | 6.57 | Cytoplasm |
| gene:MD06G1019800 | 1383 | 460 | 51020.69 | 5.23 | Cytoplasm |
| gene:MD06G1017700 | 1341 | 446 | 49846.64 | 6.48 | Cytoplasm |
| gene:MD12G1039400 | 1371 | 456 | 51076.08 | 6.99 | Cytoplasm |
| gene:MD12G1038300 | 1386 | 461 | 51244.02 | 5.83 | Cytoplasm |
| gene:MD06G1016500 | 1380 | 459 | 51567.12 | 8.42 | Cytoplasm |

**Table S2.** Activities of purified MdAAT1, MdAAT2, and MdAAT2-like proteins towards different alcohols (with Acetyl-CoA as acyl donor)

| Substrate (Alcohol) | MdAAT1 Activity (μmol/min/mg) ± SD | MdAAT2 Activity (μmol/min/mg) ± SD | MdAAT2-like Activity (μmol/min/mg) ± SD |
| --- | --- | --- | --- |
| Butanol | 185.5 ± 12.3 a | 201.2 ± 14.1 a | 45.3 ± 3.5 b |
| Hexanol | 220.8 ± 15.6 a | 245.9 ± 17.2 a | 68.7 ± 4.9 b |
| Isoamyl alcohol | 195.3 ± 13.5 a | 178.9 ± 12.8 a | 52.4 ± 4.0 b |
| 2-Methylbutanol | 165.7 ± 11.8 a | 155.2 ± 10.9 a | 48.9 ± 3.7 b |
| Benzyl alcohol | 18.5 ± 1.5 c | 22.3 ± 1.8 c | 165.8 ± 11.5 a |
| No substrate (Control) | 0 | 0 | 0 |

**Table S3.** Estimated kinetic parameters (*K_m_* and *K_cat_*/*K_m_*) of MdAAT1, MdAAT2, and MdAAT2-like proteins for key substrates

| Enzyme | Substrate | Km (mM) ± SD | Kcat (min⁻¹) ± SD | Kcat/Km (mM⁻¹ min⁻¹) ± SD |
| --- | --- | --- | --- | --- |
| MdAAT1 | Hexanol | 0.15 ± 0.01 b | 33.1 ± 1.8 a | 220.7 ± 12.5 a |
| MdAAT2 | Hexanol | 0.12 ± 0.01 b | 29.5 ± 1.6 a | 245.8 ± 13.5 a |
| MdAAT2-like | Hexanol | 0.85 ± 0.06 a | 38.2 ± 2.1 a | 44.9 ± 2.8 b |
| MdAAT1 | Butanoyl-CoA | 0.08 ± 0.01 b | 28.5 ± 1.5 a | 356.3 ± 18.2 a |
| MdAAT2 | Butanoyl-CoA | 0.10 ± 0.01 b | 30.1 ± 1.7 a | 301.0 ± 15.8 a |
| MdAAT2-like | Butanoyl-CoA | 0.05 ± 0.01 a | 32.8 ± 1.9 a | 656.0 ± 25.1 b |
| MdAAT1 | Hexanoyl-CoA | 0.25 ± 0.02 a | 25.3 ± 1.4 b | 101.2 ± 6.8 b |
| MdAAT2 | Hexanoyl-CoA | 0.22 ± 0.02 a | 28.9 ± 1.6 b | 131.4 ± 8.1 b |
| MdAAT2-like | Hexanoyl-CoA | 0.11 ± 0.01 b | 45.5 ± 2.5 a | 413.6 ± 20.5 a |

**Table S4.** Primers of TFs which have expression correlation with *MdAAT2-like* during apple fruit ripening.

| Family | Apple ID | Correlation with *MdAAT2-like* | Name | Primer-SK(5') | Primer-SK(3') |
| --- | --- | --- | --- | --- | --- |
| ARF | MD04G1096900 | 1.34 | *ARF18-like* | agaggacagcccaagctgagctcATGATTACATTTATGGATTCAAAAGAGAA | tttcagcgtaccgaattggtaccTTACATTCCTACGTTGTTGCTGC |
| G2-like | MD03G1086800 | 2.06 | *PCL1-like* | agaggacagcccaagctgagctcATGGGTGAGGAGGTAAGGATGA | tttcagcgtaccgaattggtaccTCATTTGTCATTAGAACCAGCATGT |
| G2-like | MD17G1234400 | 1.40 | *GLK1* | agaggacagcccaagctgagctcATGCTTCTTTTATCACCTTTGAGGG | tttcagcgtaccgaattggtaccTCAAGCACAGGAGGGTGGAA |
| HD-zip | MD12G1100600 | 1.80 | *ATHB-51* | agaggacagcccaagctgagctcATGGATTGGAATACCAATTTGAGAC | tttcagcgtaccgaattggtaccTTAGGGATAAGAAGGCAGATTTCC |
| HSF | MD07G1266500 | 2.18 | *B-2a-like* | agaggacagcccaagctgagctcATGGCTCCGACGCCGGTG | tttcagcgtaccgaattggtaccTCAATTGCACACCCTCTGATTAGC |
| MADS | MD02G1197400 | 2.32 | *SOC1-like* | agaggacagcccaagctgagctcATGGTGAGAGGGAAAACTCAGATG | tttcagcgtaccgaattggtaccCTAGCGCCTCGCCCTACTTT |
| MYB | MD12G1198600 | 1.27 | *MYB2-like* | agaggacagcccaagctgagctcATGGGGAGGCACTCTTGTTGC | tttcagcgtaccgaattggtaccCTAAAGGGTTTGTCCAAAAGCTACG |
| MYB | MD17G1065100 | 2.56 | *MYB27-like* | agaggacagcccaagctgagctcATGGTAGCTATGATGGGTTGGG | tttcagcgtaccgaattggtaccTTAAGCTTGTGTGTTGCTGCAAT |
| MYB | MD12G1108400 | 1.83 | *MYB58-like* | agaggacagcccaagctgagctcATGATGACTTGTCAAGCAGCCA | tttcagcgtaccgaattggtaccTTAATTCATGTCCCAGAGAAAACTTG |
| MYB | MD14G1011400 | 2.79 | *MYB60-like* | agaggacagcccaagctgagctcATGGTAAGGCCTCCTTGCTGT | tttcagcgtaccgaattggtaccTCAAACCAAGGACATTGGAGACA |
| MYB | MD02G1186900 | 1.84 | *MYB61-like* | agaggacagcccaagctgagctcATGGGTGCTCCTAAGCAGAAGTG | tttcagcgtaccgaattggtaccTCAAGAATGAGTTAACCCATGTGAG |
| MYB | MD06G1172900 | 5.14 | *MYB98-like* | agaggacagcccaagctgagctcATGGAGCTCAACAAGCTCAGAAA | tttcagcgtaccgaattggtaccTTAGTTCACTTGAGAAATCATCTCCAC |
| MYB | MD09G1188600 | 1.26 | *MYBR19* | agaggacagcccaagctgagctcATGGGAAACCAAAAGCAAAAGT | tttcagcgtaccgaattggtaccCTAAGCCAAGGCAACGACTTCA |
| WRKY | MD10G1191400 | 4.68 | *WRKY21* | agaggacagcccaagctgagctcATGGAGGAAGTTGAAGAAGCTAACA | tttcagcgtaccgaattggtaccTCATGTTGTTGTGGATTGCGA |
| WRKY | MD07G1131400 | 2.05 | *WRKY22-like* | agaggacagcccaagctgagctcATGGAGGACGATTGGGATCTTC | tttcagcgtaccgaattggtaccTCAGATACTACCAGCAGCAGTGGC |

**Table S5.** Correlation analysis of candidate transcription factors with *MdAAT2-like*.

| Family | Gene ID | Name | Correlation to *MdAAT2-like* |
| --- | --- | --- | --- |
| ARF | MD04G1096900 | *ARF18-like* | 0.8939 |
| G2-like | MD03G1086800 | *PCL1-like* | 0.8974 |
| G2-like | MD17G1234400 | *GLK1* | 0.5888 |
| HD-zip | MD12G1100600 | *ATHB-51* | 0.052 |
| HSF | MD07G1266500 | *B-2a-like* | 0.6497 |
| MADS | MD02G1197400 | *SOC1-like* | 0.5103 |
| MYB | MD12G1198600 | *MYB2-like* | 0.437 |
| MYB | MD17G1065100 | *MYB27-like* | 0.0087 |
| MYB | MD12G1108400 | *MYB58-like* | 0.6735 |
| MYB | MD14G1011400 | *MYB60-like* | 0.1913 |
| MYB | MD02G1186900 | *MYB61-like* | 0.0645 |
| MYB | MD06G1172900 | ***MYB98-like*** | 0.951 |
| MYB | MD09G1188600 | *MYBR19* | 0.6471 |
| WRKY | MD10G1191400 | ***WRKY21*** | 0.7812 |
| WRKY | MD07G1131400 | *WRKY22-like* | 0.0022 |

**Table S6.** Other primers utilized in this study.

**Part A: Gene Expression Analysis (RT-qPCR) Primers**

| Role | Primer Name | Forward primer sequence | Reverse primer sequence |
| --- | --- | --- | --- |
| RT-qPCR | MdActin | TGACCGAATGAGCAAGGAAATTACT | TACTCAGCTTTGGCAATCCACATC |
|  | MdAAT2-like | AATGTCGCACTCTTGCACTT | ACTCCAAAGCATATCCCAGT |
|  | MdMYB98-like | AGAAGTCAAATGTGGTCAAGGGTC | CGTTGCGTTCCAGTGGTTTT |
|  | MdWRKY21 | TGCCATAGAGTTCTTAGCCT | GATACATCATTTCCGTTTGC |
|  | MdAAT1 | AAGCCAACGCCTCAAGAAAC | CAGCAGCAAAGGACAACCAA |
|  | MdAAT2 | CTTGCTCCACATTTGACTTG | CATAGAGGTTCAGCCTTTGA |

**Part B: Transgene Vector Construction Primers**

| Role | Primer Name | Forward primer sequence | Reverse primer sequence |
| --- | --- | --- | --- |
| Transgene | MdAAT2-like-pC2300 | acgagctcggtaccATGATGCCATCCTCAGTACTTCAG | catggtgtcgactctagaCATCATTGACATGATCCTAGTTGATCT |
|  | MdAAT2-like-pC2301 | gcttgtcgacggatccATGATGCCATCCTCAGTACTTCAG | cgttatccataagatctACAAACAACATTCATCTTCTGCTT |
|  | MdAAT2-like-pTRV2 | gtgagtaaggttaccgaattcCAAAAATCCACTGGGATATGCTT | gagacgcgtgagctcggtaccCATCATTGACATGATCCTAGTTGATCT |
|  | MdMYB98-like-pC2300 | gagacgcgtgagctcggtaccATGGAGCTCAACAAGCTCAGAAA | catggtgtcgactctagaGTTCACTTGAGAAATCATCTCCACC |
|  | MdMYB98-like-pC2301 | gcttgtcgacggatccATGGAGCTCAACAAGCTCAGAAA | cgttatccataagatctGTTCACTTGAGAAATCATCTCCACC |
|  | MdMYB98-like-pTRV2 | gtgagtaaggttaccgaattcGTTCAGCTAACAACAACAACGGTG | gagacgcgtgagctcggtaccGTTCACTTGAGAAATCATCTCCACC |
|  | MdWRKY21-pC2300 | gagacgcgtgagctcggtaccATGGAGGAAGTTGAAGAAGCTAACA | catggtgtcgactctagaTGTTGTTGTGGATTGCGATGG |
|  | MdWRKY21-pC2301 | gcttgtcgacggatccATGGAGGAAGTTGAAGAAGCTAACA | cgttatccataagatctTGTTGTTGTGGATTGCGATGG |
|  | MdWRKY21-pTRV2 | gtgagtaaggttaccgaattcCTTTCATTTGATCGGGGCTCC | gagacgcgtgagctcggtaccTGTTGTTGTGGATTGCGATGG |

**Part C: Protein-DNA and Protein-Protein Interaction Assay Primers**
**C1: Yeast One-Hybrid (Y1H)**

| Role | Primer Name | Forward primer sequence | Reverse primer sequence |
| --- | --- | --- | --- |
| Y1H | MdMYB98-like-AD | gccatggaggccagtgaattcATGGAGCTCAACAAGCTCAGAAA | cagctcgagctcgatggatccGTTCACTTGAGAAATCATCTCCACC |
|  | MdWRKY21-AD | gccatggaggccagtgaattcATGGAGGAAGTTGAAGAAGCTAACA | cagctcgagctcgatggatccTGTTGTTGTGGATTGCGATGG |
|  | MdAAT2-like-pAbAi | atgaattgaaaagcttTAAATGAGATGTAAGGTGATATTGTGACC | gtcgacagatccccgggtaccTTCAGTAACGAGCTTAAGAACCAGC |
|  | MdWRKY21-pAbAi | atgaattgaaaagcttTATCAACGATCGTTGGTGAATAATT | gtcgacagatccccgggtaccATCTACAACAACAACCAACCACTATCC |
|  | MdMYB98-like-pAbAi | atgaattgaaaagcttGGGTCTCCGCTCCATCAATC | gtcgacagatccccgggtaccTAGAGATGCTCTAAGCAAAATGCG |

**C2: Luciferase (LUC) Reporter Assay**

| Role | Primer Name | Forward primer sequence | Reverse primer sequence |
| --- | --- | --- | --- |
| LUC | MdAAT2-like-pGreenⅡ0800-LUC | CACTATAGGGCGAATTGGGTACCTAAATGAGATGTAAGGTGATATTGTGACC | TATGTTTTTGGCGTCTTCCATGGTTCATTCAGTAACGAGCTTAAGAACCAGC |
|  | MdWRKY21-pGreenⅡ0800-LUC | CACTATAGGGCGAATTGGGTACCTATCAACGATCGTTGGTGAATAATT | TATGTTTTTGGCGTCTTCCATGGATCTACAACAACAACCAACCACTATCC |
|  | MdMYB98-like-pGreenⅡ0800-LUC | CACTATAGGGCGAATTGGGTACCGGGTCTCCGCTCCATCAATC | TATGTTTTTGGCGTCTTCCATGGTAGAGATGCTCTAAGCAAAATGCG |
|  | MdWRKY21-pGreenⅡ62-SK | agaggacagcccaagctgagctcATGGAGGAAGTTGAAGAAGCTAACA | tttcagcgtaccgaattggtaccTGTTGTTGTGGATTGCGATGG |
|  | MdMYB98-like-pGreenⅡ62-SK | agaggacagcccaagctgagctcATGGAGCTCAACAAGCTCAGAAA | tttcagcgtaccgaattggtaccGTTCACTTGAGAAATCATCTCCACC |

**C3: Electrophoretic Mobility Shift Assay (EMSA)**

| Role | Primer Name | Forward primer sequence | Reverse primer sequence |
| --- | --- | --- | --- |
| EMSA | MdAAT2-like-hot probe | AGAGGTTTCTTATTTGTAACTGTTGGATCAAATTT | AAATTTGATCCAACAGTTACAAATAAGAAACCTCT |
|  | MdAAT2-like-cold probe | AGAGGTTTCT TATTTGTAACTGTTG GATCAAATTT | AAATTTGATCCAACAGTTACAAATAAGAAACCTCT |
|  | MdAAT2-like-mutant probe | AGAGGTTTCTAAAAAAAAAAAAAAAGATCAAATTT | AAATTTGATCTTTTTTTTTTTTTTTAGAAACCTCT |
|  | MdMYB98-like-hot probe | GCAACCAACCCTGACCGTTGGATGAA | TTCATCCAACGGTCAGGGTTGGTTGC |
|  | MdMYB98-like-cold probe | GCAACCAACCAAAAAAGTTGGATGAA | TTCATCCAACTTTTTTGGTTGGTTGC |
|  | MdMYB98-like-mutant probe | GCAACCAACCAAAAAAGTTGGATGAA | TTCATCCAACTTTTTTGGTTGGTTGC |
|  | MdWRKY21-hot probe | GAGAGACTCAATTTTGTAACCGATACATAATCAAA | TTTGATTATGTATCGGTTACAAAATTGAGTCTCTC |
|  | MdWRKY21-cold probe | GAGAGACTCAAAAAAAAAAAAAAAACATAATCAAA | TTTGATTATGTTTTTTTTTTTTTTTTGAGTCTCTC |
|  | MdWRKY21-mutant probe | GAGAGACTCAAAAAAAAAAAAAAAACATAATCAAA | TTTGATTATGTTTTTTTTTTTTTTTTGAGTCTCTC |
|  | MdMYB98-like-PET32a | gccatggctgatatcggatccATGGAGCTCAACAAGCTCAGAAA | ttgtcgacggagctcgaattcGTTCACTTGAGAAATCATCTCCACC |
|  | MdWRKY21-PET32a | gccatggctgatatcggatccATGGAGGAAGTTGAAGAAGCTAACA | ttgtcgacggagctcgaattcTGTTGTTGTGGATTGCGATGG |

**Part D: Chromatin Immunoprecipitation (ChIP-qPCR) Primers**

| Role | Primer Name | Forward primer sequence | Reverse primer sequence | **Amplification Efficiency (%)** |
| --- | --- | --- | --- | --- |
| CHIP-qPCR | MdAAT2-like-S1-chip | TAAATGAGATGTAAGGTGATATTGTGACC | ACATGATTCATTCTTGTTGTATTTTATTG | **98.2** |
|  | MdAAT2-like-S2-chip | GGGGAGGTTTTTGGATGAGAA | CCCAACTTTTATCTCCTCACCCA | **101.5** |
|  | MdAAT2-like-S3-chip | GATGGAATGTCAACTTTTGTCATGG | TCCAACAGTTACAAATAAGAAACCTCTC | **95.8** |
|  | MdMYB98-like-S1-chip | GGGTCTCCGCTCCATCAATC | TTGCAAGGGAAGATCTGGTTG | **102.1** |
|  | MdMYB98-like-S2-chip | ATGCTCTTAGGGAAATAATTCTCGC | TCTTGCGCTCCCGTTTTCT | **97.6** |
|  | MdMYB98-like-S3-chip | ACCCAAAATTTGAGACCAACAAA | TTGCTTAATAGGTGGAGGAGGAA | **99.3** |
|  | MdWRKY21-S1-chip | TCAAAAAAGTTCTCATATAACAATGACC | GAAAATGGGTAAGTTGTTTTTGTCAA | **96.7** |
|  | MdWRKY21-S2-chip | ACACGAGAGACTCAATTTTGTAACCG | AGTGCAAAGACCATTAAGTCCATCT | **103.2** |
|  | MdWRKY21-S3-chip | ACCAAATTTCTATGCTGTAATGTCTTG | CCAAAACCCCAATTATTTTACCC | **98.9** |

**Part E: Protein-Protein Interaction Assay Primers**
**E1: Yeast Two-Hybrid (Y2H)**

| Role | Primer Name | Forward primer sequence | Reverse primer sequence |
| --- | --- | --- | --- |
| Y2H | MdMYB98-like-AD | gccatggaggccagtgaattcATGGAGCTCAACAAGCTCAGAAA | cagctcgagctcgatggatccGTTCACTTGAGAAATCATCTCCACC |
|  | MdWRKY21-BD | tcagaggaggacctgcatatgATGGAGGAAGTTGAAGAAGCTAACA | ccgctgcaggtcgacggatccTGTTGTTGTGGATTGCGATGG |

**E2: Bimolecular Fluorescence Complementation (BiFC)**

| Role | Primer Name | Forward primer sequence | Reverse primer sequence |
| --- | --- | --- | --- |
| BiFC | MdWRKY21-SPYCE | ggatccATGGAGGAAGTTGAAGAAGCTAACA | gtcgacTGTTGTTGTGGATTGCGATGG |
|  | MdMYB98-like-SPYNE | ggatccATGGAGCTCAACAAGCTCAGAAA | gtcgacGTTCACTTGAGAAATCATCTCCACC |

**E3: Luciferase Complementation Imaging (LCI)**

| Role | Primer Name | Forward primer sequence | Reverse primer sequence |
| --- | --- | --- | --- |
| LCI | MdMYB98-like-nLUC | cgctctagaactagtggatccATGGAGCTCAACAAGCTCAGAAA | gtcgacggtatcgataagcttGTTCACTTGAGAAATCATCTCCACC |
|  | MdWRKY21-cLUC | gggccccccctcgaggtcgacATGGAGGAAGTTGAAGAAGCTAACA | cgctctagaactagtggatccTGTTGTTGTGGATTGCGATGG |

**Part F: CRISPR/Cas9 Mutagenesis Primers**

| Role | Primer Name | Forward primer sequence | Reverse primer sequence |
| --- | --- | --- | --- |
| CRISPR/ Cas9 | MdAAT2-like-CRISPR/Cas9 | cagctagagtcgaagtagtgattgTCCAGTAAAGCCAATGCCTCgTTTCAGAGCTATGC | CTGTTTCCAGCATAGCTCTGAAAcTTTAATTTTCCAAGCTCTAAcaatcactacttcga |
|  | MdMYB98-like-CRISPR/Cas9 | cagctagagtcgaagtagtgattgCCACATTCCATTGTTTGCGTgTTTCAGAGCTATGC | CTGTTTCCAGCATAGCTCTGAAAcAAATGTTGCAGAAAACAATGcaatcactacttcga |
|  | MdWRKY21-CRISPR/Cas9 | cagctagagtcgaagtagtgattgAAACTGGAAAGGCTGTGTCTgTTTCAGAGCTATGC | CTGTTTCCAGCATAGCTCTGAAAcGGATAATCCTATATCGATTGcaatcactacttcga |

**Part G: Pull-down Assay (Recombinant Protein) Primers**

| Role | Primer Name | Forward primer sequence | Reverse primer sequence |
| --- | --- | --- | --- |
| pull-down | MdWRKY21-PET32a | gccatggctgatatcggatccATGGAGGAAGTTGAAGAAGCTAACA | ttgtcgacggagctcgaattcTGTTGTTGTGGATTGCGATGG |
|  | MdMYB98-like-PGEX4T-1 | gatctggttccgcgtggatccATGGAGCTCAACAAGCTCAGAAA | ctcgagtcgacccgggaaattcGTTCACTTGAGAAATCATCTCCACC |
